# Investigation of Electrocatalytic Methanol Oxidation Performance of Nickel Oxide Supported on Ternary CeLaCuO Nanoparticles

**DOI:** 10.1021/acsami.5c11806

**Published:** 2025-10-28

**Authors:** Louai Mahdi Maghrabi, Karthik Chimatahalli Santhakumar, Aseel G. Hussien, Smruti Medha Mishra, Dalaver Hussain Anjum, Mohammednoor Altarawneh, Ayesha Alkhoori, Kyriaki Polychronopoulou

**Affiliations:** † Department of Mechanical and Nuclear Engineering, 105955 Khalifa University of Science and Technology, Main Campus, Abu Dhabi, P.O. Box 127788, United Arab Emirates; ‡ Center for Catalysis and Separations (CeCaS), Khalifa University of Science and Technology, Main Campus, Abu Dhabi, P.O. Box 127788, United Arab Emirates; § Department of Physics, Khalifa University of Science and Technology, 127788, Abu Dhabi, United Arab Emirates; ∥ Department of Chemical and Petroleum Engineering, 11239 United Arab Emirates University, Sheikh Khalifa bin Zayed Street, Al-Ain 15551, United Arab Emirates

**Keywords:** methanol oxidation, electrocatalysis, CeLaCuO, Ni oxide supported CeLaCuO, direct methanol fuel cells

## Abstract

This study investigates the electrocatalytic activity of nickel oxide-supported CeLaCuO nanoparticles (Ni/CeLaCuO) for the methanol oxidation reaction (MOR). Nickel oxide-supported CeLaCuO nanoparticles were synthesized by a microwave method followed by wet impregnation and calcined to achieve the desired crystal structure and morphology. The materials were extensively characterized using X-ray diffraction (XRD), transmission electron microscopy (TEM), N_2_ porosimetry, Raman spectroscopy, and X-ray photoelectron spectroscopy (XPS) to confirm the morphology, surface area, pore distribution, and elemental composition of the catalysts. The electrochemical performance was assessed using cyclic voltammetry (CV), linear sweep voltammetry (LSV), chronoamperometry, and electrochemical impedance spectroscopy (EIS) in a methanol–alkaline medium environment. The results indicate that NiO decoration over the CeLaCuO support significantly enhances the MOR activity of the latter by improving charge transfer kinetics, exhibiting higher anodic current densities (52.3 mA cm^–2^) in 1.0 M CH_3_OH + 1.0 M KOH, and displaying lower charge transfer resistance due to the formation of an active NiOOH layer during oxidation. The Ni/CeLaCuO catalyst demonstrated greater stability and increased current density over extended cycles compared to that of unsupported CeLaCuO, indicating that nickel addition promotes both catalytic activity and durability. This study underscores the potential of Ni oxide-supported CeLaCuO material as a cost-effective, high-performance electrocatalyst for methanol oxidation in alkaline media, offering valuable insights for direct methanol fuel cell (DMFC) development.

## Graphical abstract

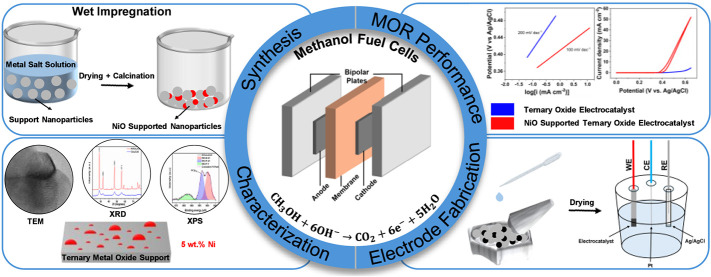

## Introduction

The methanol electrooxidation reaction (MOR) is a pivotal process in the advancement of direct methanol fuel cells (DMFCs), a technology extensively investigated since the 1950s. Despite decades of research, DMFC commercialization remains a challenge, primarily due to inefficiencies and high costs associated with the process.^1–3^ The growing interest in MOR is driven by its potential in DMFCs, which are promising candidates for both portable and stationary power applications.^3,4^ Methanol’s high energy density (332 MJ/kg), low cost^5–8^ (levelized production cost approximately falls between 642 and 795 €/t),^9^ and ease of handling make it an attractive fuel source.^5–8^ However, the complex six-electron transfer mechanism of MOR presents a significant bottleneck in DMFC performance, necessitating the development of effective electrocatalysts to overcome the associated high overpotentials and sluggish reaction kinetics.

Currently, noble metal catalysts, particularly those based on platinum (Pt), are the most effective for MOR. However, their widespread application is hindered by high costs, limited availability, and susceptibility to poisoning by reaction intermediates, such as carbon monoxide (CO).^2,4–8,10^ These challenges have motivated extensive research into alternative catalyst systems that are both cost-effective and highly efficient. Among the various approaches, alloying Pt with other metals, such as Ru and Co, has shown promise in improving catalytic activity and resistance to CO poisoning by modulating electronic structures and reducing chemisorption strength.^11–13^ Furthermore, integrating Pt with metals like Fe and Ni in PtFe and Pt/FeNi-NC catalysts has been demonstrated to enhance methanol oxidation activity and durability by facilitating toxic intermediate removal and improving electron transfer.^8,14^ Strategies such as the use of ionic liquids and carbon-based supports, including graphene oxide, have also been employed to stabilize Pt catalysts, enhance CO tolerance, and provide better anchoring for metal clusters.^15,16^ In addition to improving noble metal catalysts, significant efforts have been directed toward developing noble metal-free catalysts to address the economic and practical limitations of Pt-based systems. Noble metal-free catalysts, such as cobalt–iron layered double hydroxides, nickel-based carbides, and bimetallic nanocrystals, have emerged as promising alternatives.^17–19^ These materials offer advantages, such as lower costs, enhanced stability, and reduced susceptibility to poisoning. For example, cobalt–iron nanorods coupled with graphene oxides exhibit high electrochemical activity and stability over extended cycles due to their abundant active sites and efficient electron transfer.^19^ Similarly, nickel-based carbides leverage strong metal–carbon bonds to enhance catalytic activity while maintaining economic feasibility.^20^ Moreover, bimetallic Cu–Ni nanocrystals with yolk–shell architectures demonstrate superior durability and resistance to aggregation compared to commercial Pt/C catalysts, attributed to their structural stability and downshifted *d*-band centers that mitigate poisoning effects.^19^

Transition metal oxides represent another promising class of catalysts due to their tunable electronic structures, high thermal stability, and affordability.^11–14^ Among these, heterostructures such as NiO/NiCo_2_O_4_, which is derived from metal–organic frameworks (MOFs), have demonstrated notable electrocatalytic performance with significant current densities and enhanced electron transfer rates.^21^ Lanthanum-based metal oxides, in particular, have gained attention due to their flexible oxidation states and ability to incorporate various dopants, leading to improved catalytic activity and stability.^16^ Nickel oxide supported CeLaCuO nanoparticles present a compelling opportunity for enhancing the MOR performance. Nickel, known for its activity in alcohol oxidation reactions, can induce structural and electronic modifications in the metal oxide matrix, resulting in enhanced charge transfer and additional active sites for methanol adsorption and activation.^17,18^ Nickel doping is also expected to lower the activation energy for MOR, further boosting electrocatalytic performance.^10,19–23^

This study focuses on the synthesis and appropriate conditioning of NiO supported on CeLaCuO nanoparticles and their evaluation as electrocatalysts for the methanol oxidation reaction. While CeLaCuO alone exhibited poor electrocatalytic activity, the deposition of NiO enabled the formation of the active NiOOH phase in alkaline media, which is responsible for the high methanol oxidation activity. The CeLaCuO support provides a stable matrix that ensures efficient activation of NiO. By investigating the activity and stability of these catalysts, this work aims to improve the performance and contribute to the broader development of efficient, cost-effective catalysts for DMFCs. The insights gained will advance our understanding of non-noble-metal-based materials in electrocatalytic applications, paving the way for sustainable energy solutions.

## Experimental Section

### Materials

The metal precursors Ce­(NO_3_)_3_·6H_2_O (99.95%), La­(NO_3_)_3_·6H_2_O (99.95%), and Cu­(NO_3_)·3H_2_O (99.95%) of Sigma-Aldrich (USA) and Ni­(NO_3_)_2_·6H_2_O (Aldrich, 97.0%) were used without further purification.

### Synthesis of Ternary CeLaCuO Nanoparticles

The ternary CeLaCuO nanoparticles are synthesized as per our previously reported method.^24^ The metal precursors, cerium nitrate hexahydrate (Ce­(NO_3_)_3_·6H_2_O), lanthanum nitrate hexahydrate (La­(NO_3_)_3_·6H_2_O), and copper nitrate trihydrate (Cu­(NO_3_)_2_·3H_2_O) (all from Sigma-Aldrich, USA), were dissolved in distilled water. The molar ratios of Ce, La, and Cu were adjusted to correspond to atomic fractions of 45 at %, 45 at %, and 10 at %, respectively. The total metal loading was maintained at 0.03 mol. Ethylene glycol, used as a complexing agent, was dissolved in distilled water at a volumetric ratio of 2:1 (ethylene glycol:distilled water). The metal precursor solution was mixed with the ethylene glycol solution with continuous stirring. The mixture was subjected to microwave heating at temperatures of 130 and 170 °C. Following the reaction, the mixture was centrifuged to isolate the solid product, which was calcined in air at 500 °C for 6 h to yield the ternary CeLaCuO nanoparticles.

### Synthesis of Supported Ni Oxide Ternary CeLaCuO Nanoparticle Catalysts

Nickel nitrate hexahydrate (Ni­(NO_3_)_2_·6H_2_O, Aldrich) was dissolved in distilled deionized water to prepare the precursor solution. The solution volume was calculated to achieve a nominal Ni loading of 5 wt %. The CeLaCuO metal oxide (in powder form) was impregnated with the nickel precursor solution at 50 °C using the wet impregnation method. The mixture was thoroughly stirred to ensure uniform distribution. After impregnation, the sample was dried overnight at 120 °C. The dried solid was calcined following the methodology described in ref 24 at 500 °C for 2 h.

### Electrochemical Studies

The typical three-electrode arrangement was used for all electrochemical tests. The working electrode was made by coating ink of the synthesized material on carbon paper with slight modification of the reported literature method.^23^ To prepare catalyst ink, Ni/CeLaCuO catalyst:PVDF:carbon black were ground for 1 h in the mass ratio 20:2:3 to make a homogeneous slurry. The ink of the synthesized catalysts each containing ∼2.4 mg of Ni/CeLaCuO catalyst is allowed to dry on the surface of carbon paper (∼1.0 cm^2^). The counter electrode was a platinum rod, while the electrode used for reference was Ag/AgCl (3.5 M KCl). A Biologic VMP-3e electrochemical workstation was used to conduct voltammetric measurements. All electrochemical tests were conducted in methanol and potassium hydroxide at room temperature. Catalyst activation was done by taking multiple cycles in a specific potential window (0.0 to +0.65 V). A chronoamperometry test (*i* vs *t*) was carried out at a potential of 0.41 V to check the prolonged stability and performance of the synthesized Ni/CeLaCuO catalyst.

### Characterization Studies

The XRD analysis was conducted by using a Malvern Panalytical Empyrean diffractometer with CuKα radiation. The prepared Ni catalysts were analyzed after calcination with a diffractometer at a voltage of 45 kV and intensity of 40 mA. The X-ray diffractograms were recorded in the 2θ range of 20–80°, with increments of 0.016°. The XRD patterns were used to calculate the average crystallite size and microstrain of both samples using the Williamson–Hall (W–H) method as shown in eq 1, where *β*_*hkl*_ is the full width at half-maximum of the diffraction peak in radians, θ is the Bragg angle in radians, λ is the X-ray wavelength (0.15406 nm),^25^ *k* is the shape factor equated to 0.9,^24,25^ *ε* is the strain, and *D* is the volume-weighted crystallite size^25^ for the primary phase of the as-prepared catalysts, CeLaCuO and Ni/CeLaCuO. The Lorentz function was used to obtain *β*_*hkl*_ for each diffraction peak to plot $\frac{\beta_{hkl}\;\cos\left(\theta \right)}{k\lambda }$ vs $\frac{4\;\sin\left(\theta \right)}{k\lambda }$ as shown in Figures S1 and S2, where the slope represents the strain and the *y*-intercept represents the reciprocal of the crystallite size.

1 $$\frac{\beta_{hkl}\;\cos\left(\theta \right)}{k\lambda }=\varepsilon \left(\frac{4\;\sin\left(\theta \right)}{k\lambda }\right)+\frac{1}{D}$$

Raman spectroscopy was performed to complement the structural studies coming from powder XRD while probing the oxygen sublattice and the induced structural defects. Raman spectroscopy (Horiba JobinYvon instrument, green laser (λ = 633 nm) and 10× objective lens) was used. The elemental concentrations and chemical states on the surface were assessed through X-ray photoelectron spectroscopy (XPS) using a ThermoScientific ESCALAB QXi spectrometer equipped with a monochromated AlKα X-ray source with a photon energy of 1486.6 eV. The transmission electron microscopy (TEM) analysis was performed using a Talos 200 (Thermo Fisher Scientific) operated at 200 kV. Bright-field TEM was employed to examine the sample morphology, while high-resolution TEM (HR-TEM) provided detailed structure visualization. Selected area electron diffraction (SAED) was used for crystal structure determination, and dark-field scanning transmission electron microscopy (DF-STEM) was utilized to image the morphology in the dark-field mode. Energy-dispersive X-ray spectroscopy (EDS) enabled composition determination, and STEM-EDS was performed for elemental mapping to understand the spatial distribution of elements within the sample. The entire TEM data acquisition process was carried out using Velox V 2.5 software. Surface area and pore volume were measured by using the BET method using a 3Flex Micromeritics adsorption instrument.

## Results and Discussion

### Catalysts’ Structural and Compositional Studies

The ternary CeLaCuO nanoparticles were synthesized using microwave-assisted heating, followed by calcination at 500 °C.^24^ The wet impregnation method was employed to prepare Ni oxide-loaded CeLaCuO catalysts, with a nominal Ni loading of 5 wt %. The structural and compositional analyses confirmed the successful formation of the CeLaCuO oxide phase using XRD as shown in Figure 1a. The XRD peaks at ∼28.5°, 33.1°, 47.5°, and 56° (2θ) correspond to the (111), (200), (220), and (311) planes, respectively, of the cubic fluorite structure of CeO_2_ (JCPDS 34-0394).^15,27^ This indicates that the CeO_2_ lattice is preserved as the primary phase in both the CeLaCuO and Ni/CeLaCuO samples. A noticeable shift of diffraction peaks to smaller angles is observed after incorporating La^3+^ and Cu^2+^ cations into the lattice.^28,29^ This shift confirms the substitution of Ce^4+^ cations (0.97 Å) in the ceria lattice by larger La^3+^ cations (1.1 Å) and smaller Cu^2+^ cations (0.73 Å). Such substitution introduces strain into the lattice, altering the lattice parameters and causing the observed shift. The absence of distinct diffraction peaks associated with La_2_O_3_, CuO, or cerium–copper oxide heterophases suggests the formation of a homogeneous solid solution. The low concentration of La_2_O_3_ as a minor phase may escape detection by XRD due to its very low weight percentage in the multiphase system. The partial substitution of Ce^4+^ by La^3+^ is expected to create oxygen vacancy sites (O_v_) in the ceria lattice.^30,31^ These defects are critical for catalytic applications, as they enhance redox properties and increase catalytic activity. The XRD patterns for the Ni/CeLaCuO catalyst and CeLaCuO standalone mixed oxide support are very similar, indicating that the nickel impregnation process does not significantly alter the crystalline structure of the latter. However, changes in the peak intensity and position may reflect changes in crystallinity or lattice distortions. In addition, Ni/CeLaCuO shows low-intensity peaks that belong to NiO and CuO at ∼37.5°, 43.5°, 63.1°, and 68.5° (2θ).^24,32^

**Figure 1:**
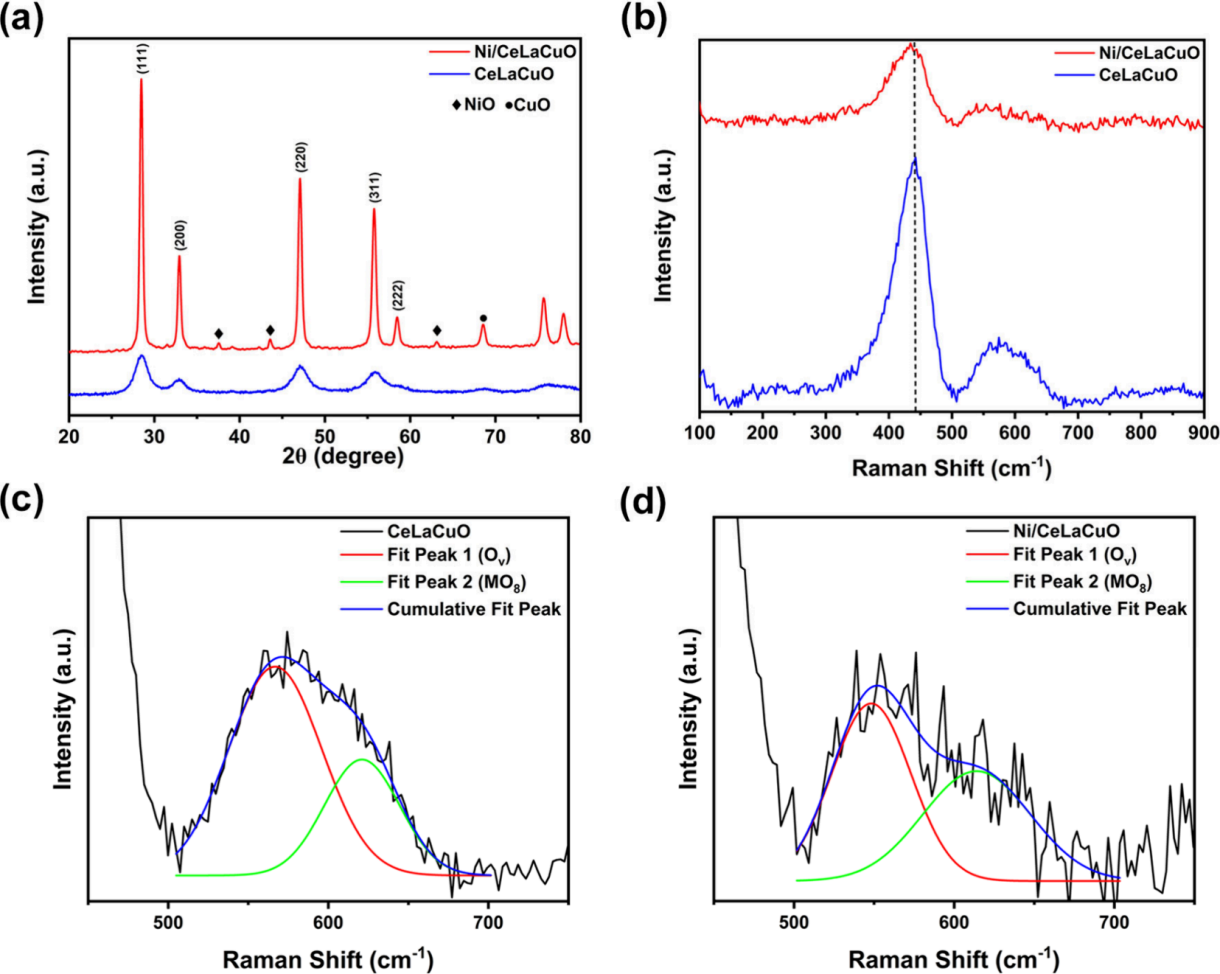
(a) Powder XRD patterns of CeLaCuO and Ni/CeLaCuO, (b) Raman spectra of CeLaCuO and Ni/CeLaCuO, (c) deconvolution of the Raman spectra between 500 and 700 cm^–1^ for CeLaCuO, and (d) deconvolution of the Raman spectra between 500 and 700 cm^–1^ for Ni/CeLaCuO.

The Raman spectra provide insight into the structural characteristics of CeLaCuO and Ni/CeLaCuO samples, as shown in Figure 1b. The prominent band near 460 cm^–1^ is attributed to the F_2g_ mode, which is characteristic of the cubic fluorite structure of ceria (CeO_2_).^33^ This peak corresponds to the symmetric stretching of oxygen atoms around the cerium ions, indicating that the ceria lattice is preserved in both CeLaCuO and Ni/CeLaCuO samples. This observation aligns with the XRD results. The broadening of the F_2g_ peak in both CeLaCuO and Ni/CeLaCuO suggests the introduction of lattice distortions caused by doping with La^3+^ and Cu^2+^ ions. These distortions are associated with the substitution of Ce^4+^ cations (0.97 Å) by larger La^3+^ cations (1.1 Å) and smaller Cu^2+^ cations (0.73 Å), creating strain in the lattice. A slight redshift (toward lower wavenumbers) of the F_2g_ peak is observed in the Ni/CeLaCuO sample compared to CeLaCuO.^24,15^ This shift is attributed to additional lattice distortions induced by the incorporation of Ni species through either substitution or surface interaction. The appearance of a weak shoulder or broad band in the region of 400–600 cm^–1^ indicates the presence of oxygen vacancies (O_v_). These vacancies arise from the partial substitution of Ce^4+^ by La^3+^ and Cu^2+^, as well as Ni incorporation. Oxygen vacancies are critical for enhancing the redox properties and the catalytic performance of the material. The I_O_v__/I_F_2g__ intensity ratio can help indicate the concentration of oxygen vacancies in both materials where a larger ratio means a greater vacancy concentration.^24^ The calculated ratios obtained from Figures 1(b–d) are 0.219 and 0.185 for CeLaCuO and Ni/CeLaCuO, respectively.

Figures 2a and 2c reveal nanoparticles with irregular shapes and a polycrystalline nature that is much more intense in the CeLaCuO catalyst, as further highlighted in Figures 2b and 2d. The coalesced particle size for the Ni/CeLaCuO catalyst is in the nanometer range (20–40 nm), as highlighted in Figure 2c, confirming the nanoscale morphology of the synthesized material. The latter is in agreement with the calculated crystallite size via W–H analysis estimated as 33.01 nm. The HR-TEM images in Figures 2b and 2d reveal well-defined lattice fringes, with spacings of 0.315, 0.324, and 0.328 nm for CeLaCuO and 0.325 nm for Ni/CeLaCuO, determined via FFT and IFFT analysis. All spacings correspond to the (111) plane of the CeO_2_ phase and are consistent with previous XRD and Raman characterizations shown in Figure 1.

**Figure 2:**
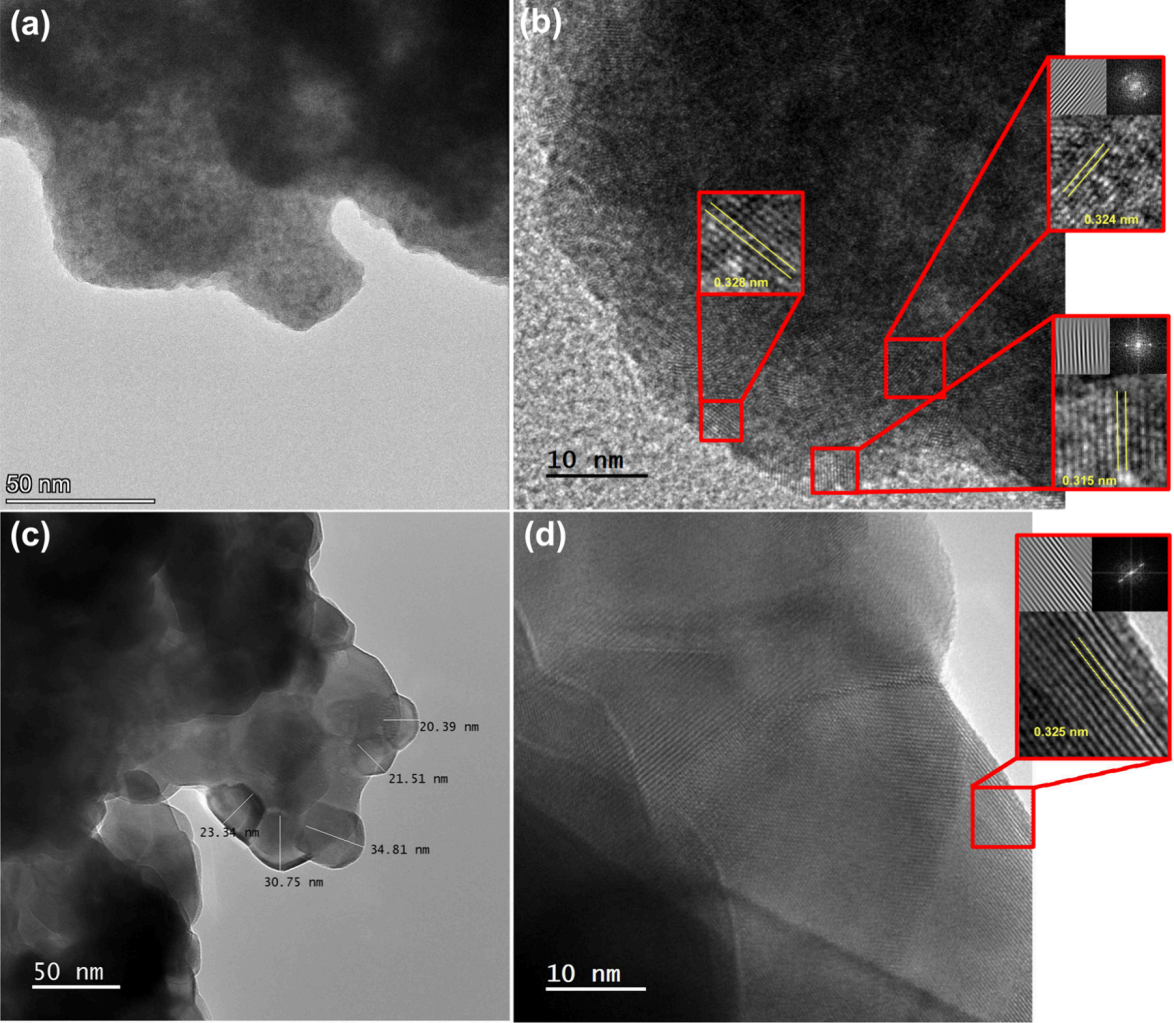
(a) TEM image of CeLaCuO, (b) HR-TEM image of CeLaCuO, (c) TEM image of Ni/CeLaCuO, and (d) HR-TEM image of Ni/CeLaCuO.

The SAED ring patterns shown in Figures S4 and S5 further confirm the latter analysis, with the undoped CeLaCuO nanoparticles having a higher degree of polycrystallinity in comparison to the Ni/CeLaCuO catalyst. The latter is also confirmed in Figure 1a by the narrow sharp peaks exhibited in the case of Ni/CeLaCuO in comparison to the broader peaks of CeLaCuO. The interplanar spacing (*d*-spacing) values reported in Table S1 are consistent with the literature and the HR-TEM analysis, corresponding to the cubic fluorite structure of CeO_2_ in both CeLaCuO and Ni/CeLaCuO, while additional phases such as NiO are present in the Ni/CeLaCuO catalyst. The interplanar spacings obtained from SAED analysis were assigned to their respective Miller indices CeO_2_ (111) and NiO ((311) and (222)), confirming the presence of these phases in the catalysts. The elemental mapping images shown in Figure 3 demonstrate the dispersion of Ce, La, Cu, and O in CeLaCuO (Figure 3(b–f)) and Ce, La, Cu, O, Ni in Ni/CeLaCuO (Figure 3(h–l)).

**Figure 3:**
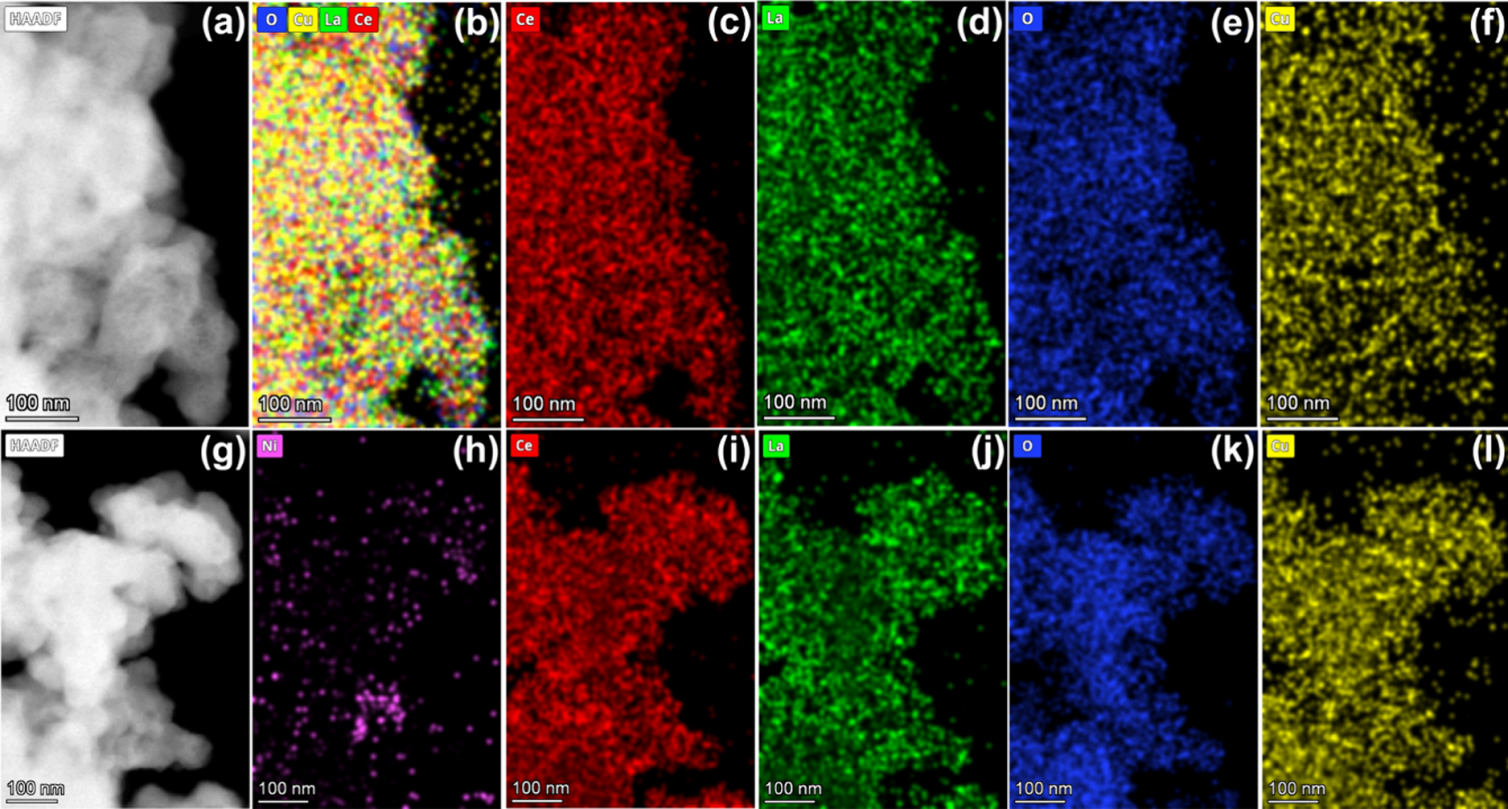
High-angle annular dark field (HAADF) of (a) CeLaCuO and TEM-EDS elemental mapping images of (b) combined elements O, Cu, La, and (c) Ce, (d) La, (e) O, and (f) Cu in the as-prepared CeLaCuO catalyst. HAADF of (g) Ni/CeLaCuO and TEM-EDS imaging of (h) Ni, (i) Ce, (j) La, (k) O, and (l) Cu in the as-prepared Ni/CeLaCuO catalyst.

The X-ray photoelectron spectroscopy (XPS) analysis provides detailed information about the composition of CeLaCuO and Ni/CeLaCuO by measuring the binding energies of various core-level electrons. The spectra shown in Figures S10 and S13 correspond to the binding energies for key elements in the catalyst: La (La 3d), Ce (Ce 3d), O (O 1s), Cu (Cu 2p), and Ni (Ni 2p). For CeLaCuO and Ni/CeLaCuO, the XPS spectra show the presence of La^3+^ (∼833 eV and ∼ 853 eV correspond to La 3d_5/2_ and La 3d_3/2_) and Ce^3+^/Ce^4+^ (∼882 eV and ∼900 eV are attributed to Ce 3d_5/2_ and Ce 3d_3/2_) oxidation states.^34^ The copper species are predominantly in the Cu^2+^ oxidation state, as shown in Figures 4a and 4c, which is typical for copper oxide and/or copper in oxide environment catalysts. The oxygen species are primarily in the form of metal–oxygen bonds, indicating a stable oxide structure.^35^ In the Ni/CeLaCuO composite, the incorporation of Ni is confirmed by the presence of Ni^2+^ and Ni^3+^ in the XPS spectrum.^36^ In both CeLaCuO and Ni/CeLaCuO, the XPS data reveal oxygen vacancies (O_v_) through the O 1s spectrum, where the peak around 531 eV indicates the presence of oxygen vacancy-related species, as shown in Figures 4b and 3d. The existence of Ce^3+^ (from the Ce 3d spectrum) also supports the presence of oxygen vacancies in the ceria lattice. The Ce^3+^ species are stabilized in the presence of oxygen vacancies, which are essential for the redox properties of ceria.^37,38^ The oxygen vacancy sites (O_v_) enhance the catalytic performance of the materials by providing active sites for the adsorption and activation of molecules, making these materials more effective for electrocatalytic reactions.

**Figure 4:**
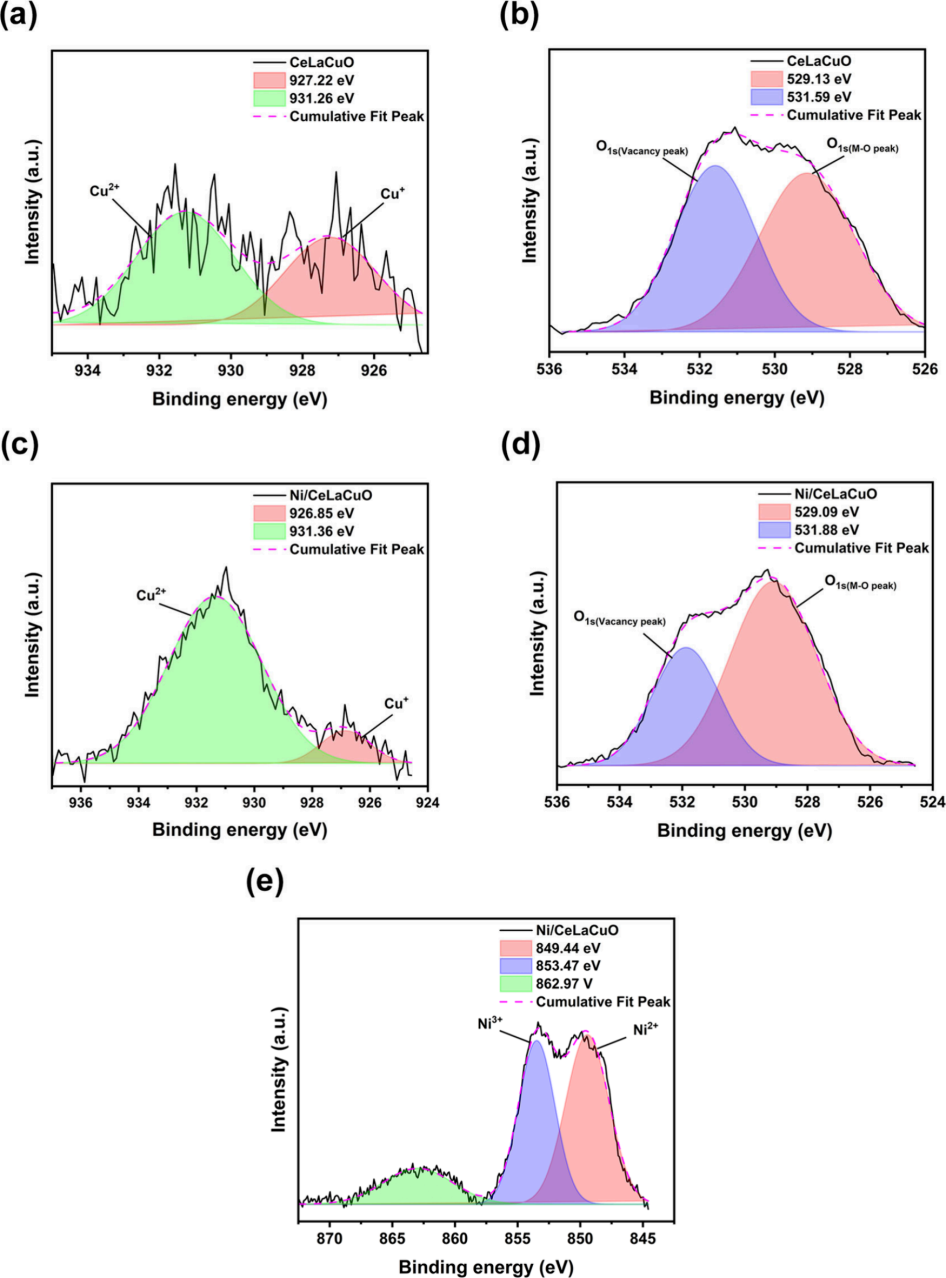
Deconvoluted XPS spectra of (a) Cu 2p and (b) O 1s for CeLaCuO nanoparticles and (c) Cu 2p, (d) O 1s, and (e) Ni 2p for Ni/CeLaCuO nanoparticles.

The Brunauer–Emmett–Teller (BET) results shown in Figure 5 for CeLaCuO and Ni/CeLaCuO provide essential insights into the surface area, pore structure, and pore volume of these materials, which are critical factors for evaluating their suitability as electrocatalysts for methanol oxidation reactions. The adsorption isotherms for both CeLaCuO and Ni/CeLaCuO show a typical Type IV isotherm (Figure 5a), which is characteristic of mesoporous materials (pores in the range 2–50 nm). The sharp increase in adsorbed volume at higher relative pressures (*p*/*p*_0_) indicates the capillary condensation of nitrogen within the mesopores, which is a hallmark of mesoporous materials.^24,39^ The CeLaCuO sample shows a higher maximum volume adsorbed at high relative pressures, while Ni/CeLaCuO shows a lower adsorption volume, indicating that the incorporation of nickel oxide into the material may have a slight effect on decreasing the adsorption capacity. For example, at a relative pressure (*p*/*p*_0_) of around 1, the volume absorbed is 16.72 cm^3^ g^–1^, while for Ni/CeLaCuO, the measured volume absorbed is 12.11 cm^3^ g^–1^. The pore volume vs pore width graphs (Figure 5b) show the distribution of pore sizes for both catalysts, indicating that both CeLaCuO and Ni/CeLaCuO have well-developed mesopores. The CeLaCuO catalyst showed a higher cumulative pore volume due to the incorporation of nickel in Ni/CeLaCuO and a higher BET surface area of 9.83 m^2^ g^–1^ in comparison to 4.92 m^2^ g^–1^. The increase in pore volume with increasing pore width suggests that the materials are predominantly mesoporous with accessible pore channels for both reactant and product movement during the electrocatalytic process. The presence of these mesopores ensures the efficient diffusion of reactants such as methanol into the catalyst, while the well-developed mesopore structure of both CeLaCuO and Ni/CeLaCuO helps mitigate mass transport limitations, a common challenge in electrochemical reactions.

**Figure 5:**
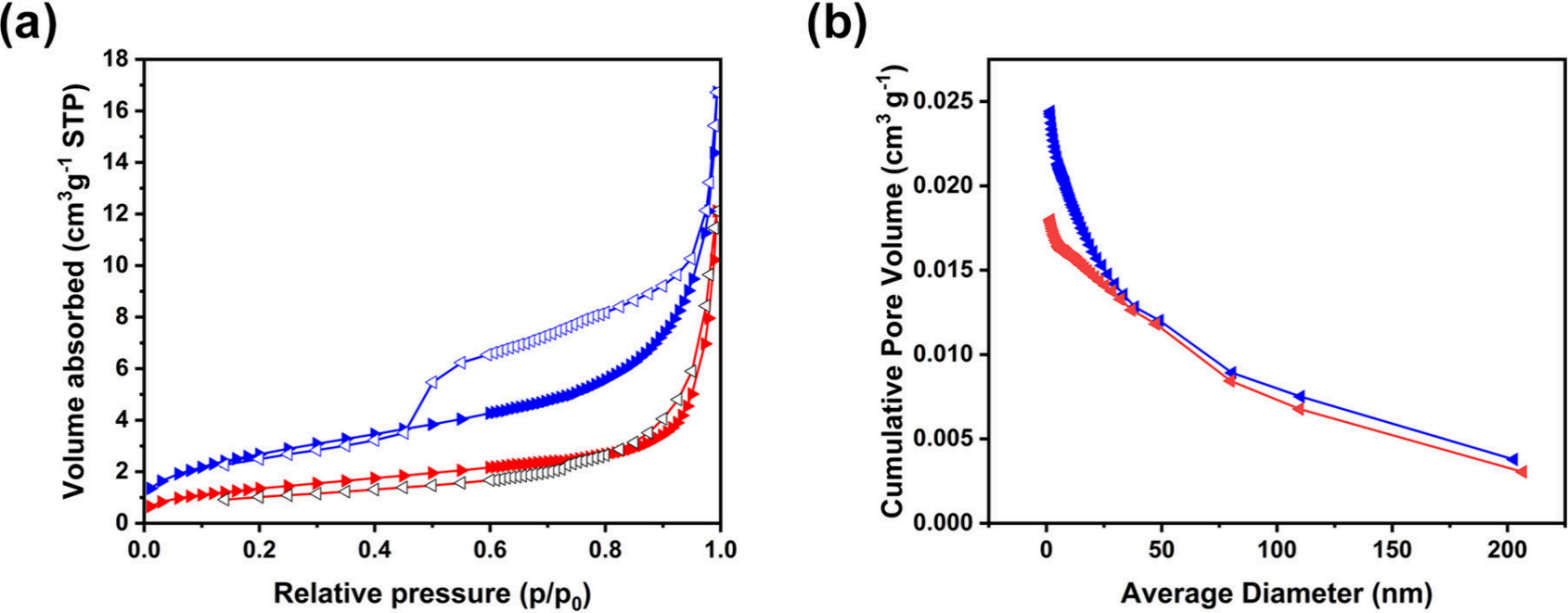
(a) N_2_ adsorption–desorption isotherms obtained at 77 K for the CeLaCuO (blue) and Ni/CeLaCuO (red) nanoparticles; (b) the cumulative pore volume (cm^3^ g^–1^) (inset) for the CeLaCuO (blue) and Ni/CeLaCuO (red) nanoparticles.

The synthesized ternary CeLaCuO and Ni/CeLaCuO nanoparticles exhibit structural and compositional features that make them highly promising for electrocatalytic applications. The incorporation of La^3+^ and Cu^2+^ cations induces lattice strain, 0.00733 and 0.00205 for the as-prepared catalysts CeLaCuO and Ni/CeLaCuO, respectively, which can enhance catalytic activity by generating oxygen vacancies (O_v_).^40^ These vacancies are critical for electrocatalytic reactions, as they improve the material’s redox properties, facilitating charge transfer and enhancing the material’s redox cycling.^41,42^ The oxygen vacancies also play an important antipoisoning role in methanol electrooxidation, as they promote the adsorption of OH^–^ and accelerate the oxidative removal of the adsorbed CO.^43^ Thus, in turn, it boosts the overall electrocatalytic performance. Additionally, the interaction between Ni and the ceria/ceria-related phase(s) is key to improving charge transfer and potentially increasing electrocatalytic activity.^44^ The calculated microstrains of both catalysts demonstrate significant lattice distortion. However, the Ni/CeLaCuO catalyst shows a relatively smaller value, attributed to the double calcination process applied during the synthesis procedure. Nickel is well-known for its catalytic properties in electrooxidation reactions, and its dispersion or incorporation into the ceria matrix may create synergistic effects with CeLaCuO, leading to improved electrocatalytic behavior for methanol oxidation.^45^ The ability to tune the electronic structure through doping and defect introduction allows for optimization of methanol adsorption and activation, making these materials suitable for efficient electrocatalysis.

## Electrochemical Oxidation of Methanol

### Electrocatalytic Surface Activation and Evaluation Parameters

To evaluate the methanol oxidation reaction (MOR) performance of the synthesized catalysts, a three-electrode system was employed, as shown in Figure 6a. The synthesized catalysts were activated using cyclic voltammetry in a 1.0 M KOH electrolyte. Figure S14 presents a cyclic voltammetry (CV) profile of CeLaCuO, recorded in 1.0 M KOH at a scan rate of 50 mV/s where no significant current density was observed after 100 cycles. Under similar conditions, the experiment was conducted on the Ni/CeLaCuO electrocatalyst over multiple cycles to assess the activation and stability of the material under repeated electrochemical cycling. The CV curves corresponding to cycles 25, 75, and 100 reveal a gradual increase in the current with increasing cycle number. At cycle 25, the current response remains relatively low, suggesting that the material is still stabilizing. However, as the number of cycles progresses to 75 and 100, the current gradually increases, indicating an activation phenomenon as shown in Figure S15. This activation can arise from surface restructuring or improved electrolyte interaction, enhancing the catalytic properties of the material over time. The introduction of nickel to the CeLaCuO catalyst introduces clear redox peaks, particularly in the potential range between 0.3 and 0.5 V vs Ag/AgCl, corresponding to redox processes of nickel species (Ni^2+^/Ni^3+^). From the early stages, as shown by the curve at cycle 25 in Figure S15, the nickel component actively participates in the electrochemical reaction. The redox peaks become more pronounced by cycle 75, reflecting an improvement in the catalytic activity through activation. By cycle 100, anodic and cathodic currents increase further, underscoring the material’s capability to enhance its catalytic performance by gradually developing a thicker catalytically active NiOOH layer as shown in eq 2.^46,47^ Methanol electrooxidation on Ni-based anodes is believed to proceed according to the mechanism presented in eq 3, generating carbonate, formaldehyde, formic acid, CO, and CO_2_ as possible intermediates or end products.^48^

2 $$\mathrm{NiO}+{\mathrm{OH}}^{-}↔\mathrm{NiOOH}+e^{-}$$

3 $$\mathrm{NiOOH}+{\mathrm{CH}}_{3}\mathrm{OH}\to \mathrm{Ni}{\left(\mathrm{OH}\right)}_{2}+\mathrm{products}$$

**Figure 6:**
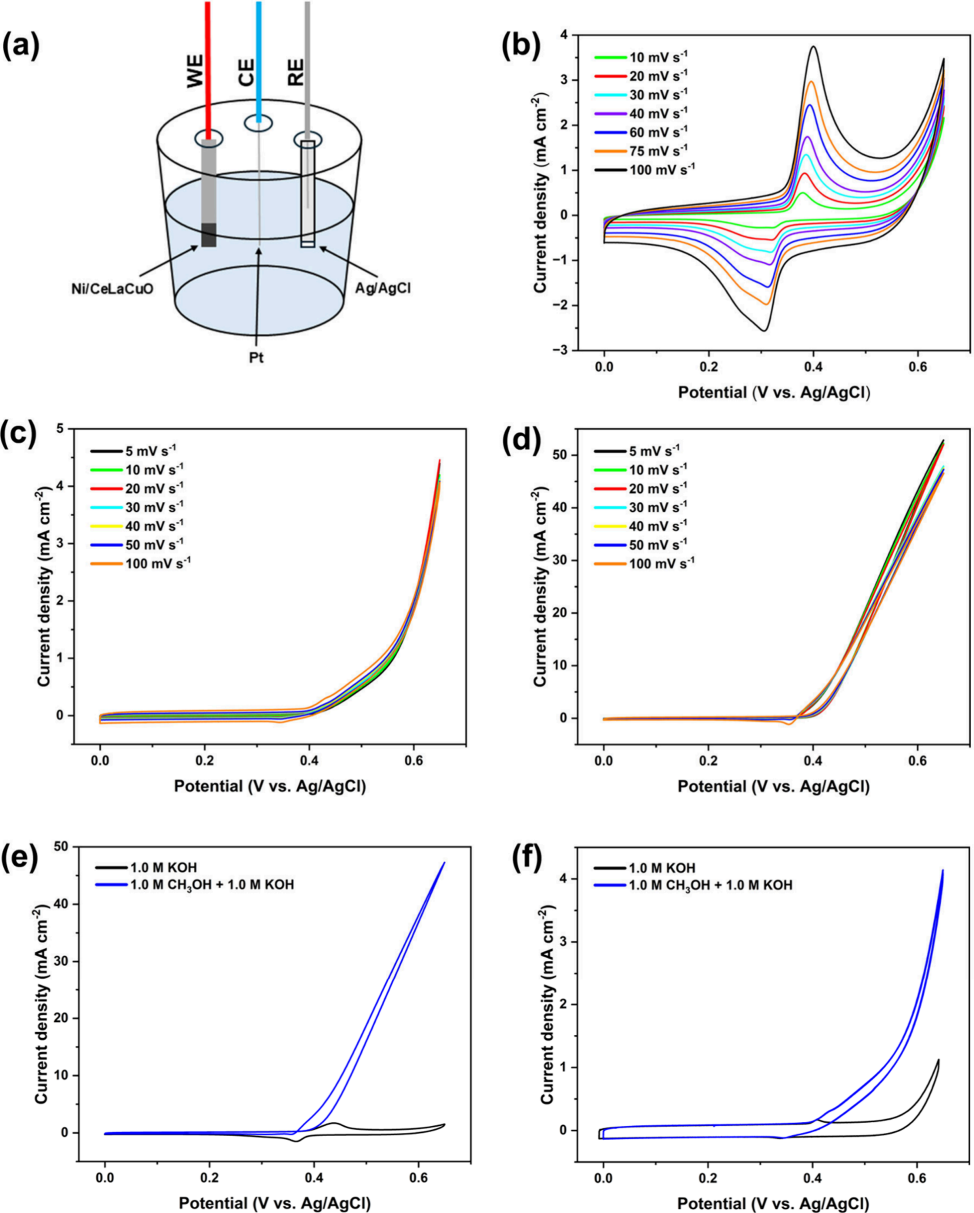
Electrochemical evaluation in the three-electrode system with 1.0 M KOH and 1.0 M CH_3_OH: (a) three-electrode setup schematic, (b) CV curves of the Ni/CeLaCuO electrocatalyst at varying scan rate (10–100 mV s^–1^) in 1.0 M KOH, (c) CV curves of the CeLaCuO electrocatalyst at varying scan rate (5–100 mV s^–1^) in 1.0 M KOH + 1.0 M CH_3_OH, (d) CV curves of the Ni/CeLaCuO electrocatalyst at varying scan rate (5–100 mV s^–1^) in 1.0 M KOH + 1.0 M CH_3_OH, (e) comparison of CV curves for the Ni/CeLaCuO electrocatalyst in 1.0 M KOH and 1.0 M KOH + 1.0 M CH_3_OH at a scan rate of 50 mV s^–1^, (f) comparison of CV curves for the CeLaCuO electrocatalyst in 1.0 M KOH and 1.0 M KOH + 1.0 M CH_3_OH at a scan rate of 100 mV s^–1^.

These observations indicate that nickel incorporation not only stabilizes the catalyst but also boosts its electrocatalytic activity, particularly through the formation of a NiOOH layer, which is essential for efficient methanol oxidation. The difference in redox peak potentials (Δ*E*_p_) can help evaluate how efficiently electrons move between the electrode surface and the active sites.^49^ Δ*E*_p_ was calculated as 0.0878 V vs Ag/AgCl for the Ni/CeLaCuO electrocatalyst from the CV profile shown in Figure S15 at the 100th cycle. Compared to the values reported by Abdullah et al.,^49^ the electron transfer kinetics are significantly faster. Nonetheless, the electrochemical reaction of the formation of the NiOOH layer is known to be a diffusion-controlled process as previously reported in refs 49 and 50. The latter is confirmed by plotting the peak current density against the square root of the scan rate as described by the Randles–Sevcik relation^49^ and obtaining a linear relationship. As shown in Figure S16, an almost perfect fit is obtained for both the anodic and cathodic peak current density plots.

Figure S17 shows cyclic voltammetry (CV) profiles of CeLaCuO recorded in 1.0 M KOH at varying scan rates of 10, 20, 30, 40, 60, 75, and 100 mV s^–1^. The cyclic voltammogram highlights CeLaCuO catalyst’s electrochemical response under different scan rates, with a progressive increase in current observed as scan rate increases. The low scan rates yield smaller current responses, indicating mass transport of participating species toward the electrode surface is the limiting factor for current, thereby indicating the process as diffusion-controlled. Higher scan rates yield larger currents, consistent with the expected dependence of diffusion-controlled processes on scan rate.^51^ The absence of distinct redox peaks however suggests that the current response of CeLaCuO mainly arises from nonfaradaic processes or weak electrochemical interactions with the electrolyte.

Figure 6b presents the CV profiles for Ni/CeLaCuO under identical experimental conditions, demonstrating a stronger electrochemical response compared with the unsupported catalyst. The addition of nickel oxide introduces clear redox peaks, particularly within the potential range of 0.3–0.5 V vs Ag/AgCl, which correspond to the reversible oxidation and reduction of nickel species. As scan rate increases, both anodic and cathodic peak currents grow, indicating fast charge transfer and good electrode kinetics. The higher current densities and more defined redox peaks across all scan rates suggest that nickel enhances catalytic activity of the CeLaCuO matrix. For CeLaCuO and Ni-supported CeLaCuO, scan rates of 100 mV/s and 50 mV/s were applied, respectively, as shown in Figures 6e and 6f. CeLaCuO displays a moderate anodic current in the positive potential range, reflecting its inherent catalytic properties. However, the inclusion of 5 wt % Ni significantly enhances catalytic response, with higher anodic currents observed across the potential range. The nickel oxide-supported variant also exhibits characteristic redox peaks associated with nickel oxidation and reduction processes, confirming nickel’s active role in promoting methanol oxidation.^52^ This comparison indicates the superior catalytic ability of Ni-supported CeLaCuO for methanol electro-oxidation in alkaline media.

Figures 6c and 6d demonstrate cyclic voltammetry profiles for CeLaCuO and Ni/CeLaCuO electrodes, respectively, in a methanol–alkaline electrolyte (1.0 M CH_3_OH + 1.0 M KOH) at a scan rate of 5, 10, 20, 30, 40, 50, and 100 mV s^–1^. The profiles capture the electrochemical response of each electrode, revealing significant differences in peak currents and redox behavior, particularly between 0.3 and 0.65 V. The CV curve of CeLaCuO displays modest anodic currents, suggesting that while it catalyzes methanol oxidation, activity is moderate. However, with 5 wt % Ni incorporation, the Ni/CeLaCuO electrode exhibits markedly higher anodic and cathodic currents, indicating an enhanced electrochemical response due to the catalytic role of Ni in promoting methanol oxidation. This comparison highlights that nickel oxide addition to the CeLaCuO matrix notably improves its catalytic efficiency in alkaline methanol solutions, as further demonstrated in Figure 7a as a direct comparison between the two profiles. In Figures S19–S23, CV profiles for Ni/CeLaCuO are presented for different methanol concentrations (0.5, 2.0, 4.0, and 8.0 M CH_3_OH) in 1.0 M KOH, with measurements taken at a constant scan rate of 20 mV s^–1^ in Figure S19 and varying scan rates in Figures S20–S23. Increasing the methanol concentration from 0.5 to 2.0 M leads to a significant increase in anodic current densities, indicating enhanced methanol oxidation activity. At lower concentrations, the response is subdued, likely due to limitations in availability of methanol at the electrode surface. As concentration increases, the availability of methanol improves, leading to higher currents and showcasing the material’s catalytic efficiency at various methanol concentrations up to a certain limit. Figure S19 shows a slight decrease in the current as the concentration of methanol goes up to 4.0 M CH_3_OH; however, a major decrease in the current takes place when the concentration of methanol is set to 8.0 M, where the values become comparable to 0.5 M. This analysis underscores versatility of the Ni/CeLaCuO catalyst in promoting methanol oxidation across a range of concentrations. This progressive increase in current density indicates that CeLaCuO is capable of efficient catalytic activity across a wide range of methanol concentrations, though the anodic currents are generally lower compared to Ni-supported variants, highlighting the enhancement imparted by nickel on catalytic performance.^53,54^

**Figure 7:**
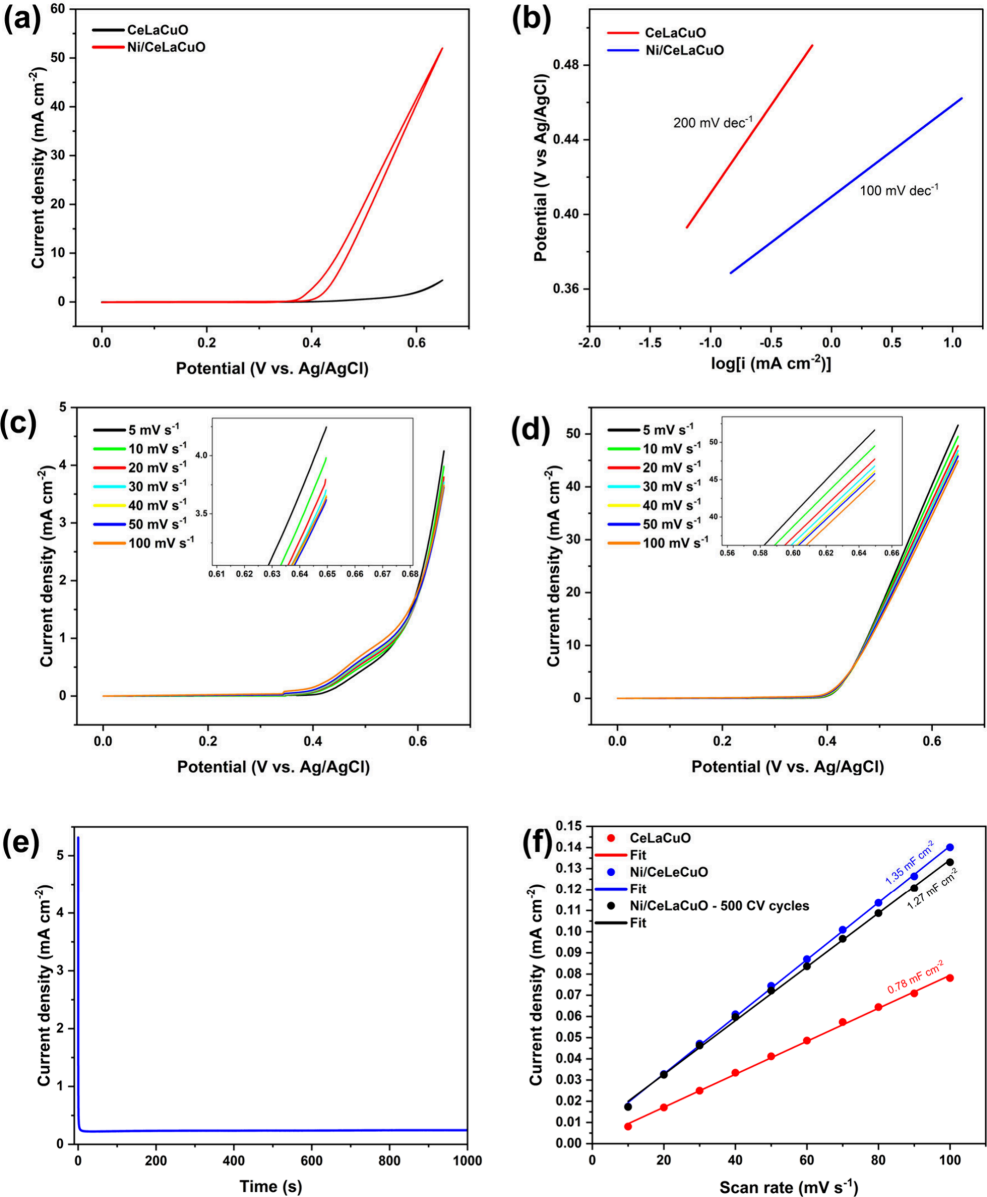
(a) CV curves of CeLaCuO and Ni/CeLaCuO recorded in a 1.0 M KOH + 1.0 M CH_3_OH electrolyte at a scan rate of 20 mV s^-1^, (b) Tafel slopes of CeLaCuO and Ni/CeLaCuO in a 1.0 M KOH + 1.0 M CH_3_OH electrolyte, (c) LSVs of CeLaCuO with different scan rates (5–100 mV s^–1^) in a 1.0 M KOH + 1.0 M CH_3_OH electrolyte, (d) LSVs of Ni/CeLaCuO with different scan rates (5–100 mV s^–1^) in a 1.0 M KOH + 1.0 M CH_3_OH electrolyte, (e) chronoamperometric curves recorded in the 1.0 M KOH + 1.0 M CH_3_OH electrolyte at 0.41 V for the Ni/CeLaCuO electrocatalyst, (f) linearly fitted curves of the double-layer capacitance in 1.0 M KOH + 1.0 M CH_3_OH for CeLaCuO and Ni/CeLaCuO electrocatalysts at 0.15 V vs Ag/AgCl.

### Electrochemical Performance Studies

The Tafel slope is a descriptor that helps analyze electrochemical reaction kinetics by quantifying the overpotential required to increase the current density by 10-fold. It provides insights into the charge transfer coefficient, the number of electrons involved, and the potential changes in the reaction mechanism. Variations in the Tafel slope indicate shifts in the rate-determining step of the reaction.^55^ Figure 7b shows the Tafel slope of CeLaCuO and Ni/CeLaCuO, which are 100 and 200 mV dec^–1^, respectively, which is indicative that nickel oxide-supported material exhibits more favorable charge transfer kinetics than unsupported CeLaCuO. Figures 7c and 7d present the LSV curves of CeLaCuO and Ni/CeLaCuO electrodes in a methanol–alkaline medium (1.0 M CH_3_OH + 1.0 M KOH) with varying scan rates of 5, 10, 20, 30, 40, 50, and 100 mV s^–1^. Both materials show oxidation peaks, but the Ni/CeLaCuO electrode demonstrates significantly higher anodic current densities compared to CeLaCuO, underscoring improved catalytic activity conferred by nickel addition. Increased current density of Ni-supported CeLaCuO suggests an efficient catalytic pathway for methanol oxidation, as nickel facilitates enhanced electron transfer and stabilization of intermediates during oxidation. This comparison highlights the key role of nickel in enhancing methanol oxidation on CeLaCuO-based electrodes. Figure 7e shows the chronoamperometry results for Ni/CeLaCuO in a solution of 1.0 M CH_3_OH + 1.0 M KOH at an applied potential of 0.41 V. The current response is recorded over time to assess the stability of the electrode during methanol oxidation. Initially, a high current density is observed, which gradually stabilizes over time. This decay in current is typical for methanol oxidation due to formation of intermediate species on the electrode surface that can inhibit catalytic activity.^52^ However, a relatively stable current over an extended period reflects good stability of Ni/CeLaCuO, suggesting its robustness as an anodic material for methanol oxidation. Figure S24 presents the EIS data, represented by Nyquist plots, for CeLaCuO and Ni/CeLaCuO electrodes in a solution of 1.0 M CH_3_OH + 1.0 M KOH. The semicircular region in each plot corresponds to charge transfer resistance at the electrode/electrolyte interface. The Ni/CeLaCuO electrode shows a smaller semicircle (0.34 Ω cm^2^) compared to CeLaCuO (0.56 Ω cm^2^), indicating a lower charge transfer resistance due to the presence of nickel, which enhances the electron transfer rate. The reduced resistance in the Ni-supported material suggests that nickel not only boosts methanol oxidation activity but also facilitates faster charge transfer kinetics, thus improving the overall electrochemical performance. An increase of the double-layer capacitance (*C*_dl_) indicates a larger electrochemical active surface area (ECSA) since $\mathrm{ECSA}=\frac{C_{\mathrm{dl}}}{C_{s}}$ . Another electrocatalytic performance descriptor,^56,57^ *C*_dl_ is obtained by plotting the current density $\frac{j_{a}-j_{c}}{2}$ against the scan rates where *j*_a_ is the anodic current density and *j*_c_ is the cathodic current density as $C_{\mathrm{dl}}=\frac{\frac{j_{a}-j_{c}}{2}}{v}$ .^57^ Cyclic voltammetry sweeps in the nonfaradaic region between 0.0 and 0.2 V vs Ag/AgCl for scan rates between 10 and 100 mV s^–1^ are shown in Figures S25, S26, and S27 for CeLaCuO and Ni/CeLaCuO, respectively; a higher scan rate results in a profile that encapsulates higher current densities. Figure 7f shows the calculated *C*_dl_ values of the CeLaCuO and Ni/CeLaCuO electrocatalysts at 0.15 V vs Ag/AgCl, reported as 0.78 and 1.35 mF cm^–2^, respectively, indicating a higher exposure of active sites for the Ni-incorporated electrocatalyst and, thus, an enhanced catalytic activity. Additionally, the double-layer capacitance of Ni/CeLaCuO measured after 500 CV cycles is 1.27 mF cm^–2^, indicating only a minor decrease. The mass loading of Ni/CeLaCuO was evaluated by cyclic voltammetry at 20 mV s^–1^ after catalyst activation in 1.0 M KOH at 50 mV s^–1^. The Ni/CeLaCuO electrocatalyst was subjected to 1000 consecutive CV cycles to evaluate its durability, as presented in Figure S28. During the initial cycles, the anodic peak current gradually increased, reaching a maximum at approximately the 40th cycle. Beyond this point, a progressive decline in the current was observed over the course of cycling. After completing 1000 cycles at a scan rate of 20 mV s^–1^, the electrocatalyst retained 71.3% of its maximum peak current. A loading range of 1 to 4.8 mg was investigated, as shown in Figure S29. The lowest (1 mg) and highest (4.8 mg) loadings did not provide optimal activity toward methanol oxidation. In contrast, a loading of approximately 3 mg exhibited the best performance, with a peak current density of 52 mA cm^–2^. A slight increase to 3.4 mg further improved the current density by about 8%.

## Conclusions

In this study, ternary CeLaCuO nanoparticles were synthesized through microwave-assisted heating and calcined at 500 °C, followed by the wet impregnation method to prepare Ni oxide loaded CeLaCuO catalysts with 5 wt % Ni. Comprehensive structural and compositional characterization confirmed the formation of a homogeneous CeO_2_-based solid solution, with La and Cu substituting Ce in the lattice and generating oxygen vacancies that enhance the redox properties of the material. The incorporation of nickel oxide further improved the lattice strain and facilitated the formation of a NiOOH layer, which is critical for efficient methanol oxidation. Electrochemical analysis demonstrated that the addition of nickel oxide significantly enhanced the electrocatalytic performance of the CeLaCuO system. CV and LSV experiments revealed higher current densities and well-defined redox peaks for the Ni/CeLaCuO electrode compared to those of CeLaCuO, indicating better charge transfer and catalytic activity. The chronoamperometric analysis showed that the Ni-supported material exhibited good stability during methanol oxidation, while EIS data revealed a reduced charge transfer resistance for the Ni/CeLaCuO electrode, suggesting improved electron transfer kinetics. The results underscore the superior electrocatalytic performance of the Ni/CeLaCuO catalyst for methanol oxidation, demonstrating its potential as an effective and stable anode material in fuel cell applications. This study highlights the importance of tuning the electronic structure and incorporating transition metals such as Ni to enhance the catalytic behavior of ceria-based materials in energy conversion processes. The findings provide a pathway for designing advanced electrocatalysts with optimized performance for practical electrochemical applications.

## Supplementary Material



## Notes

Ni/CeLaCuO synthesis schematic; W–H fitting of CeLaCuO and Ni/CeLaCuO; SAED analysis of CeLaCuO and Ni/CeLaCuO; EDS elemental mapping of Ni/CeLaCuO; XPS spectra of CeLaCuO and Ni/CeLaCuO; CV of CeLaCuO and Ni/CeLaCuO in KOH at different cycles; fitting of peak current density vs square root of scan rate for Ni/CeLaCuO; CV of CeLaCuO in 1.0 M KOH at different scan rates; LSV of CeLaCuO vs Ni/CeLaCuO in 1.0 M KOH + 1.0 M CH_3_OH; CV of Ni/CeLaCuO in different CH_3_OH concentrations; fitted Nyquist plots of CeLaCuO and Ni/CeLaCuO; CV of CeLaCuO and Ni/CeLaCuO in nonfaradaic region; peak current density retention of Ni/CeLaCuO; CV of different Ni/CeLaCuO ink mass loadings (PDF)
