# Supplementary material for: Investigation of Electrocatalytic Methanol Oxidation Performance of Nickel Oxide Supported on Ternary CeLaCuO Nanoparticles
Source: ACS Appl Mater Interfaces. 2025 Oct 28;17(45):61955–67. doi: 10.1021/acsami.5c11806 (PMC12616601; doi:10.1021/acsami.5c11806)
Supplement: Supplementary file 1 [file am5c11806_si_001.pdf]

Supporting Information

Investigation of Electrocatalytic Methanol Oxidation Performance of Nickel Oxide Supported on Ternary CeLaCuO Nanoparticles

Louai Mahdi Maghrabi[1,2], Karthik

[1]*Department of Mechanical and Nuclear Engineering, Khalifa University of Science and Technology, Main Campus, Abu Dhabi, P.O. Box 127788, United Arab Emirates*

[2]*Center for Catalysis and Separations (CeCaS), Khalifa University of Science and Technology, Main Campus, Abu Dhabi, P.O. Box 127788, United Arab Emirates*

[3]*Department of Physics, Khalifa University of Science and Technology, 127788, Abu Dhabi, United Arab Emirates*

[4]*United Arab Emirates University, Department of Chemical and Petroleum Engineering, Sheikh Khalifa bin Zayed Street, Al-Ain 15551, United Arab Emirates*

*Corresponding Email: Kyriaki.polychrono@ku.ac.ae

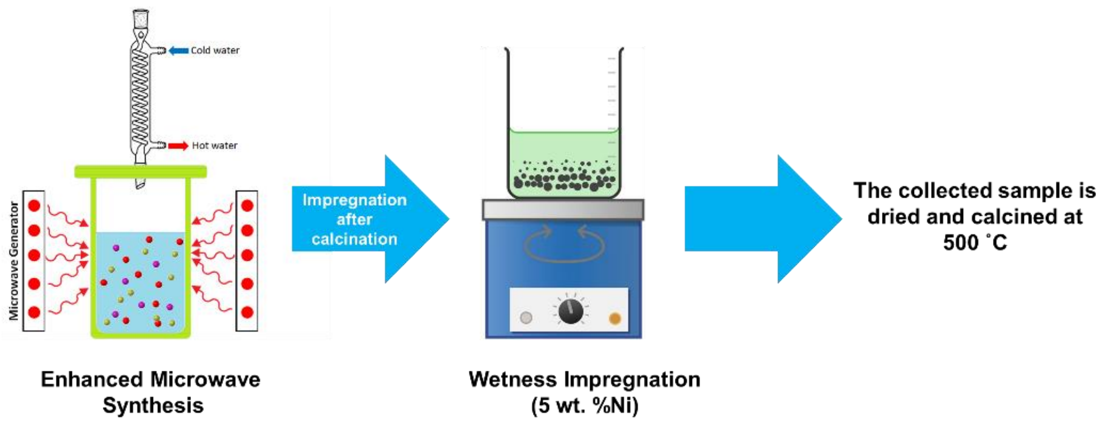
Cold water
Hot water
Microwave Generator
Impregnation
after
calcination
The collected sample is
dried and calcined at
500 ˚C
Enhanced Microwave
Synthesis
Wetness Impregnation
(5 wt. %Ni)

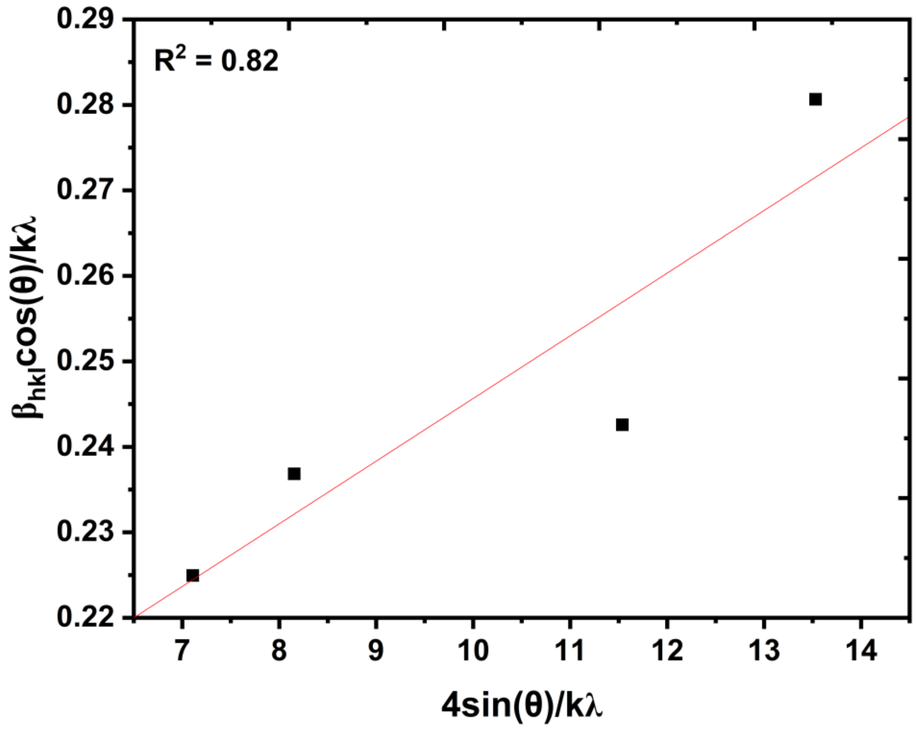
R2 = 0.82
βhklcos(θ)/kλ
4sin(θ)/kλ
0.29
0.28
0.27
0.26
0.25
0.24
0.23
0.22
7
8
9
10
11
12
13
14

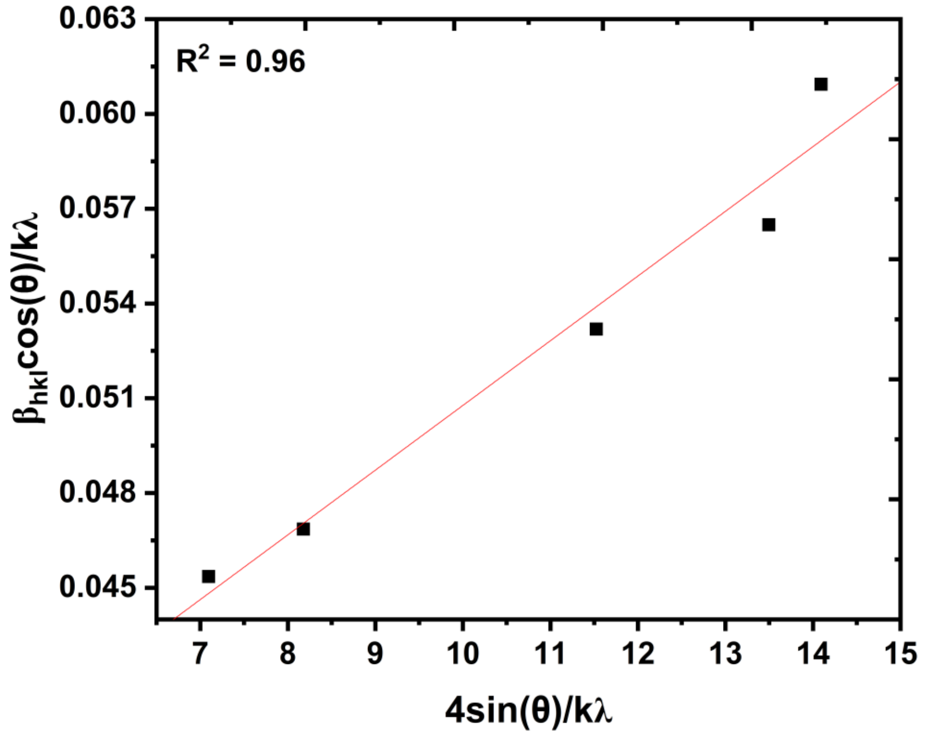

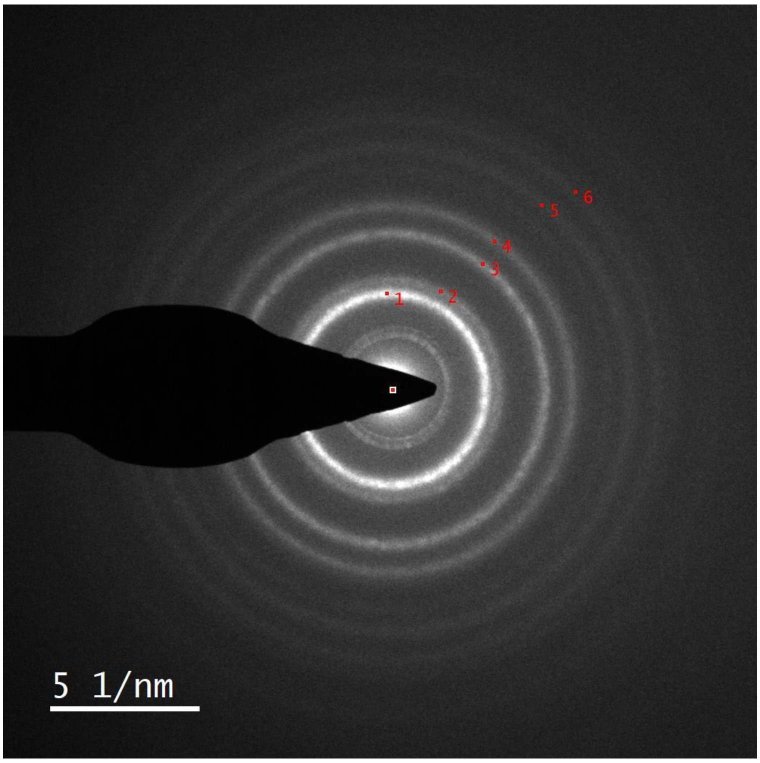

Figure S4. Selected Area Electron Diffraction (SAED) pattern with marked (1–6) lattice fringes for

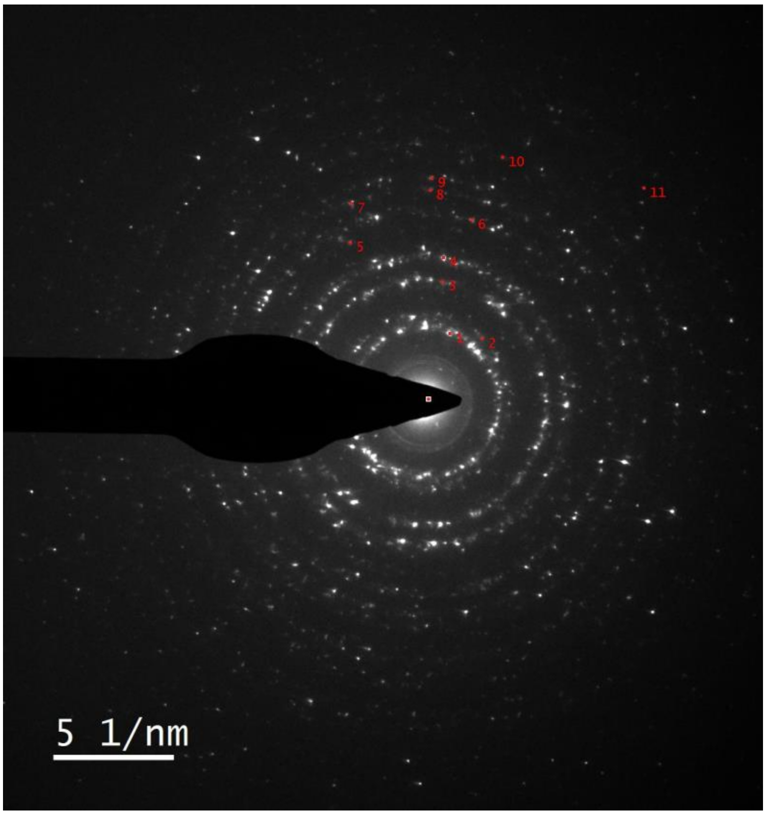

Selected Area Electron Diffract

Table S1. d-spacing and the assigned miller-index based on the SAED spots/rings in Figure S4 and S5.

Spot	d-spacing (nm)	Species	Miller -index	d-spacing (nm) from literature	Reference
			CeLaCuO		
**1**	0.312	CeO_2	(1 1 1)	0.314	[1]
**2**	0.275	CeO_2	(2 0 0)	0.273	[1]
**3**	0.195	-	-	-	-
**4**	0.167	CeO_2	(3 1 1)	0.164	[1]
**5**	0.127	-	-	-	-
**6**	0.112	-	-	-	-
			Ni/CeLaCuO		
**1**	0.350	CeO_2	(1 1 1)	0.314	[1]
**2**	0.297	$Ni(OH)_2$	$(1\ \bar{1}\ 0)$	0.290	[2]
**3**	0.204	NiO	(3 1 1)	0.210	[3]
**4**	0.170	-	-	-	-
**5**	0.138	-	-	-	-
**6**	0.131	NiO	(2 2 2)	0.121	[4]
**7**	0.115	-	-	-	-
**8**	0.116	-	-	-	-
**9**	0.109	-	-	-	-

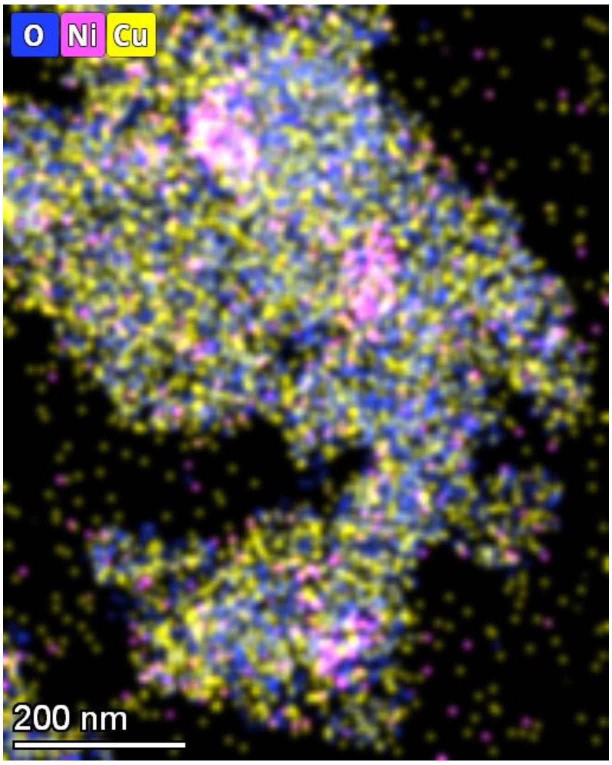

Figure S6. EDS elemental mapping image of O, Ni, and Cu from the Ni/CeLaCuO nanoparticles.

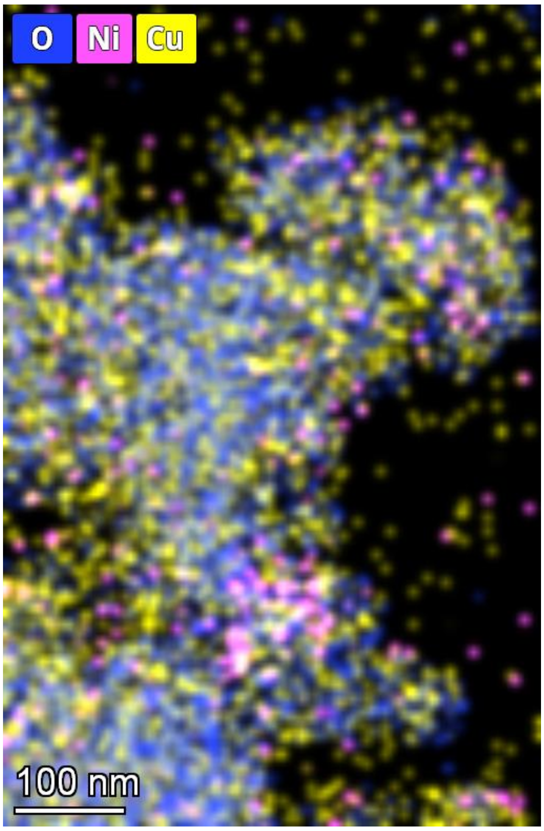

Figure S7. EDS elemental mapping image of O, Ni, and Cu from the Ni/CeLaCuO nanoparticles.

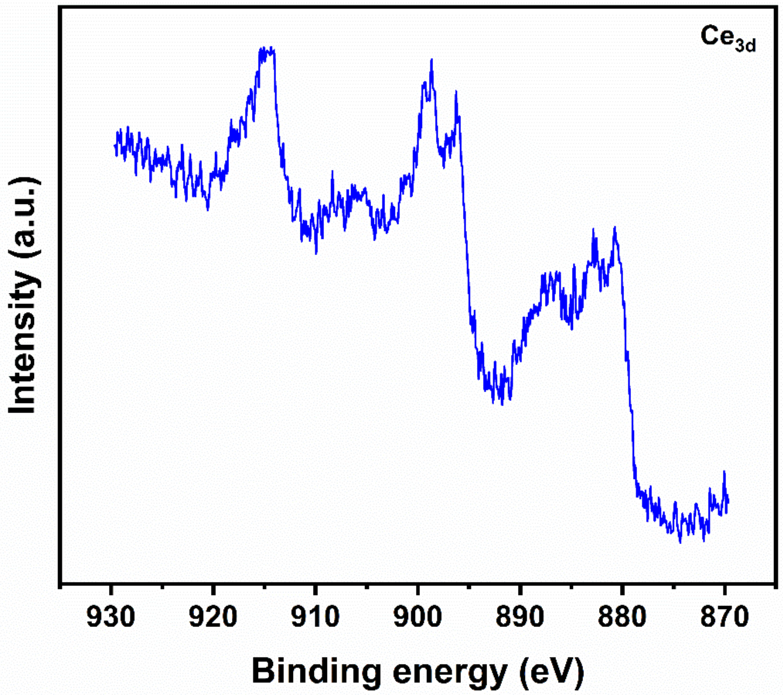

Figure S8. XPS spectra of Ce *3d* in CeLaCuO nanoparticles.

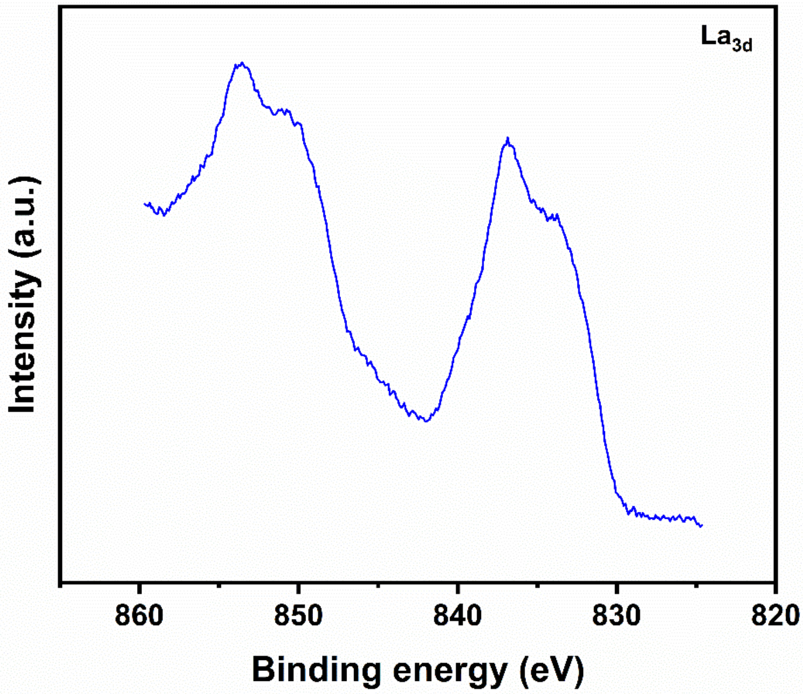

Figure S9. XPS spectra of La *3d* in CeLaCuO nanoparticles.

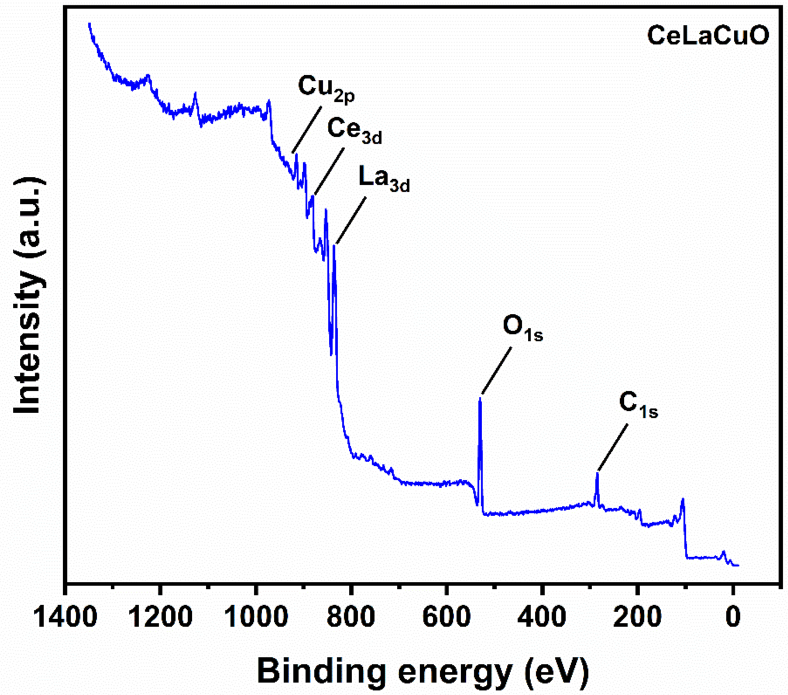

Figure S10. Total XPS spectra of CeLaCuO nanoparticles.

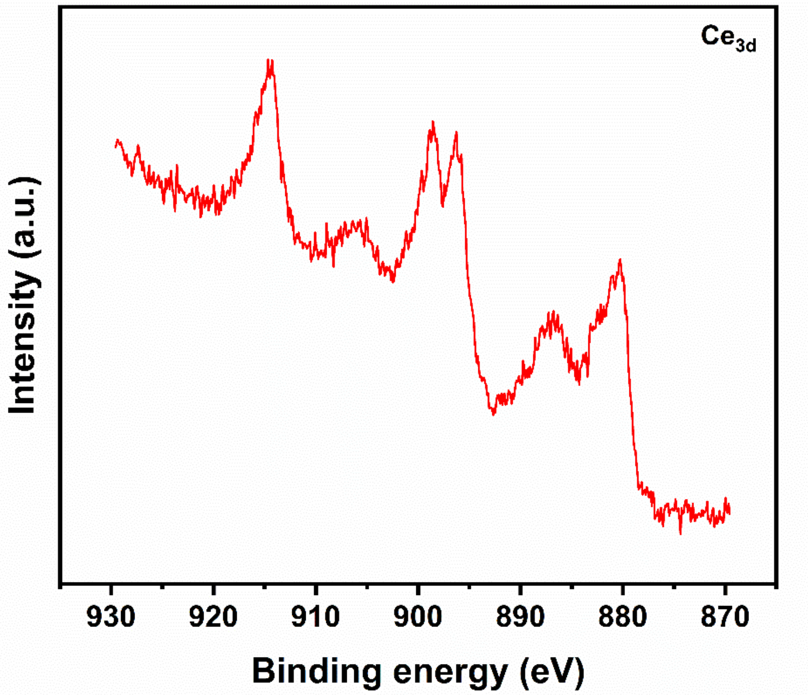

Figure S11. XPS spectra of Ce *3d* in Ni/CeLaCuO nanoparticles.

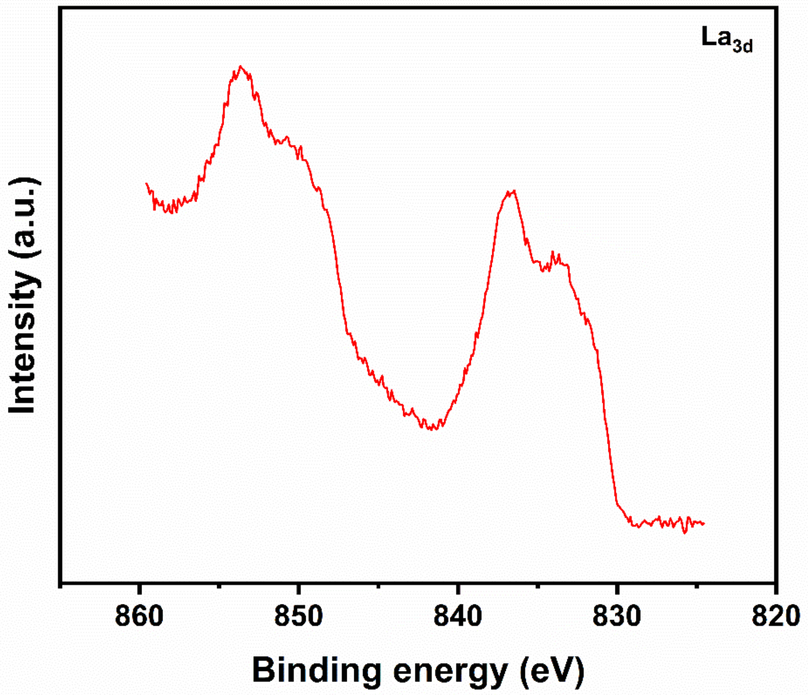

Figure S12. XPS spectra of La *3d* in Ni/CeLaCuO nanoparticles.

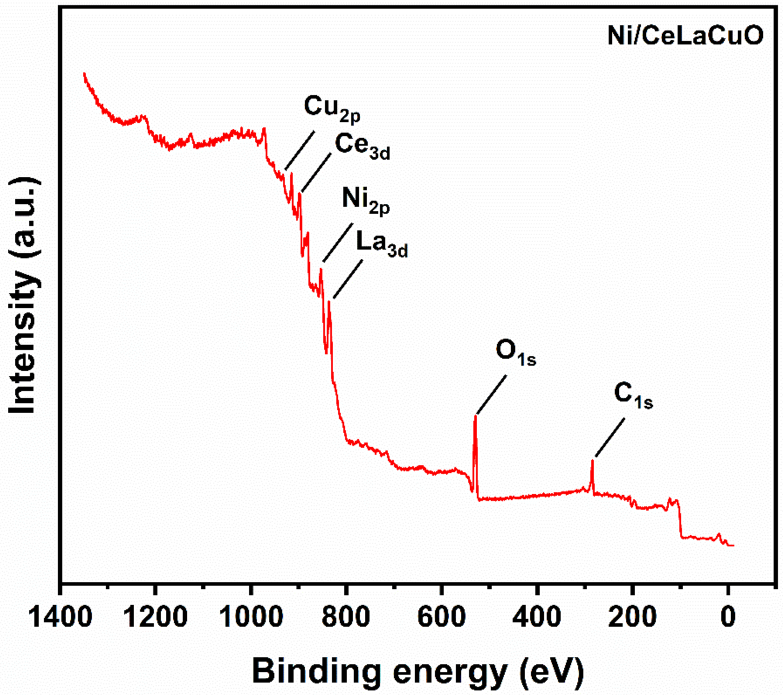

Figure S13. Total XPS spectra of Ni/CeLaCuO nanoparticles.

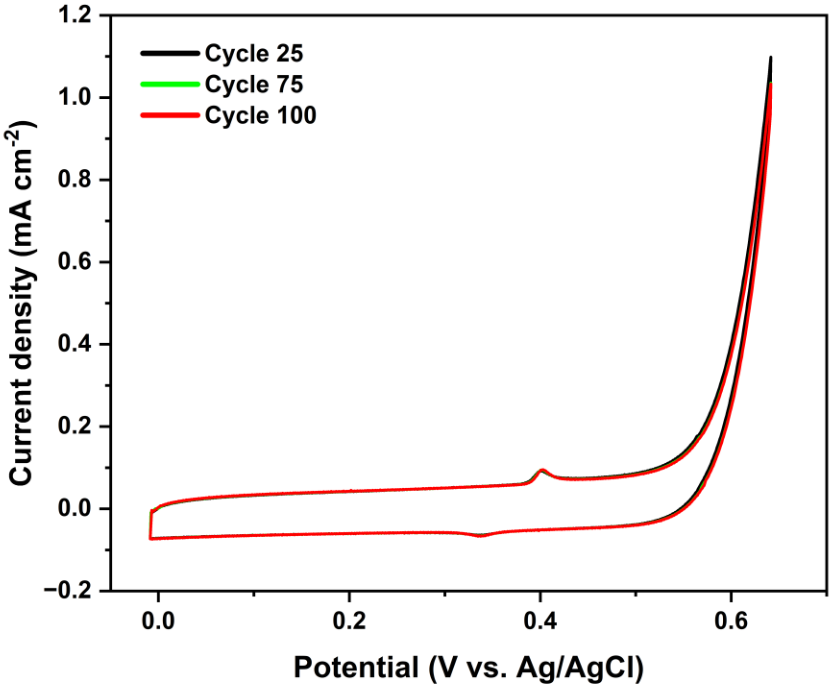

Figure S14. CV curves at different cycles of CeLaCuO electrocatalyst at a scan rate of 50 mV s^{-1} in 1.0 M KOH.

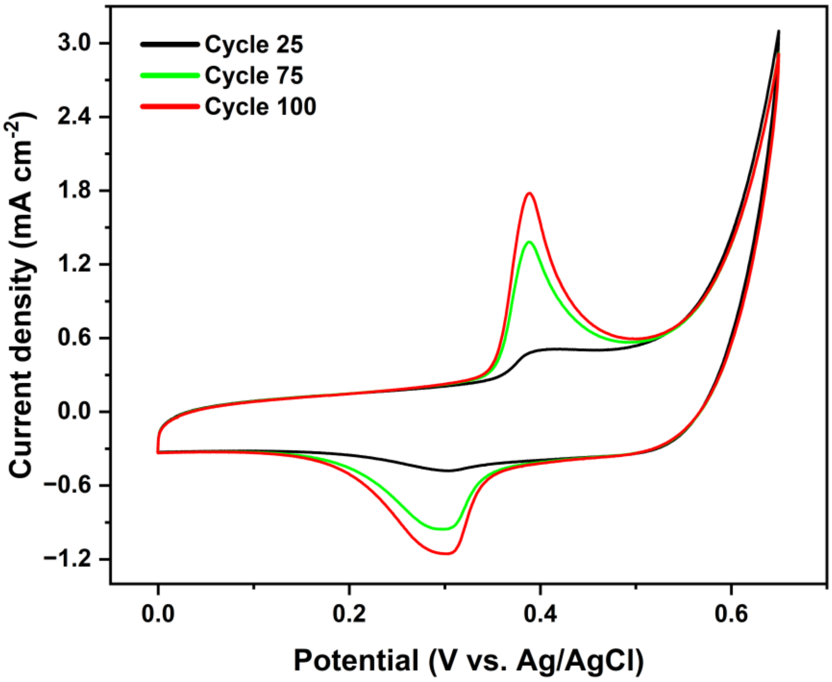

Figure S15. CV curves at different cycles of Ni/CeLaCuO electrocatalyst at a scan rate of 50 mV s^{-1} in 1.0 M KOH.

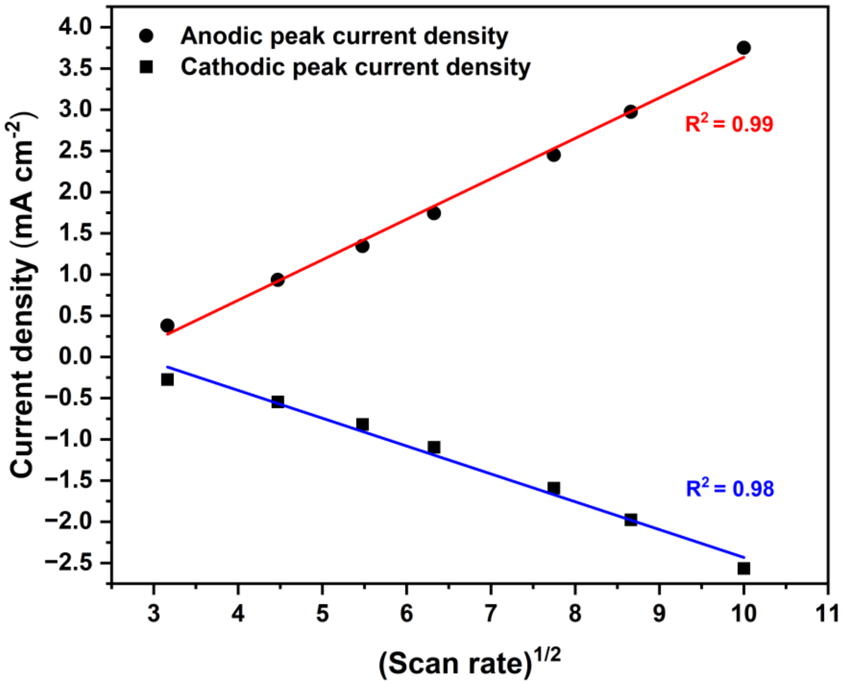

Figure S16. The anodic and cathodic peak current densities vs. the square root of the scan rate for Ni/CeLaCuO electrocatalyst in 1.0 M KOH.

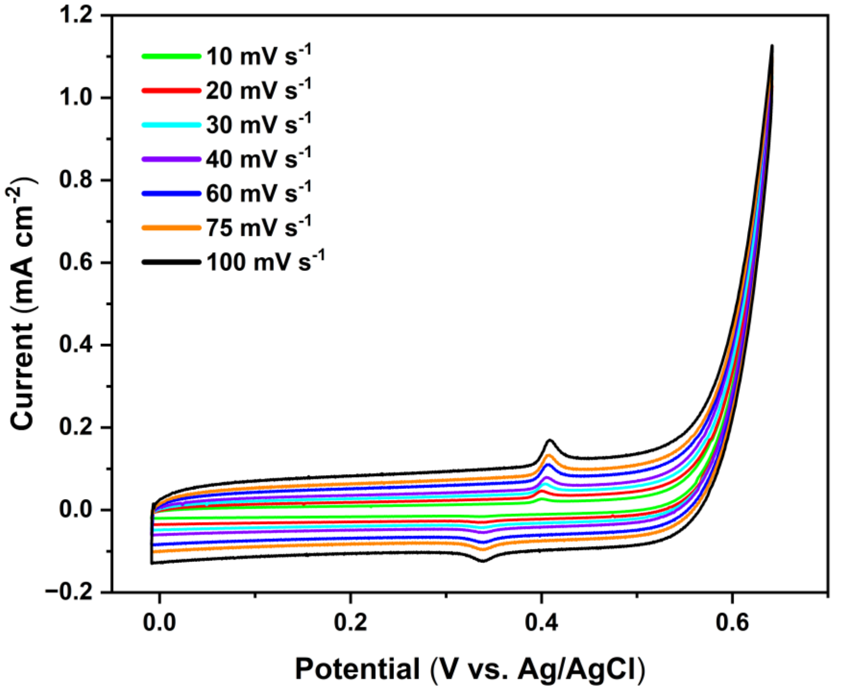

Figure S17. CV curves of CeLaCuO electrocatalyst at varying scan rates (10 mV s^{-1} - 100 mV s^{-1}) in 1.0 M KOH.

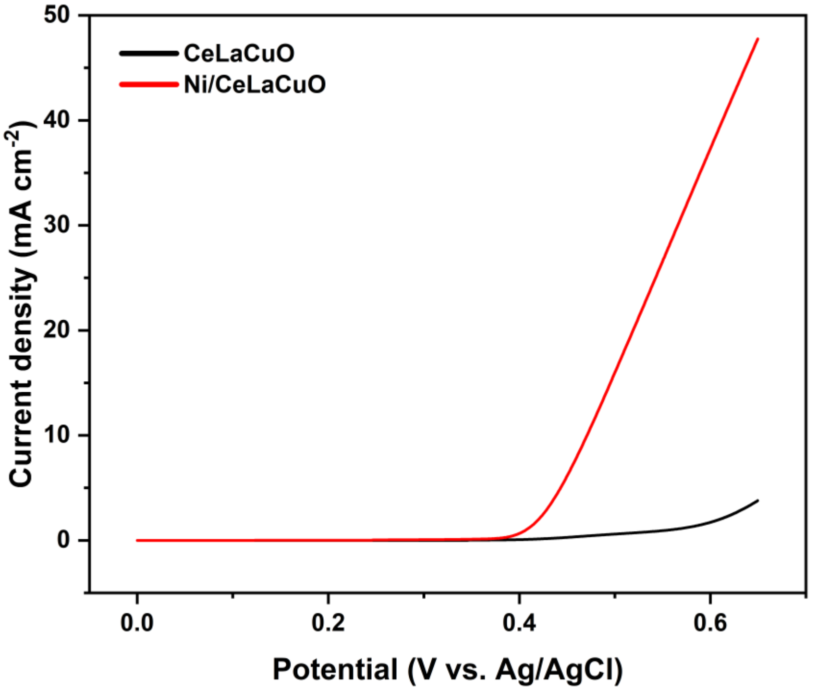

Figure S18. LSVs of CeLaCuO and Ni/CeLaCuO at a scan rate of 20 mV s^{-1} in a 1.0 M KOH + 1.0 M CH_3OH electrolyte.

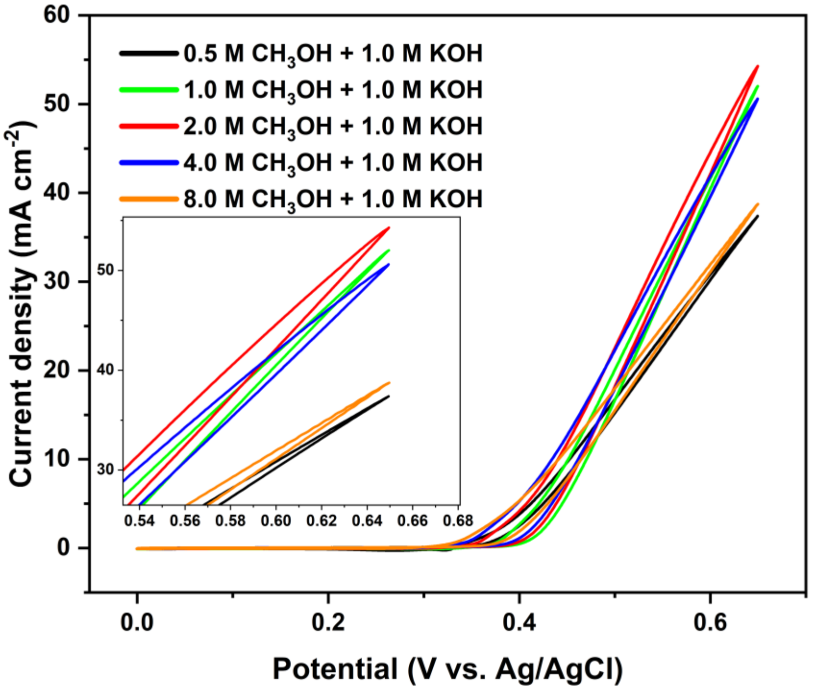

Figure S19. CVs of Ni/CeLaCuO at a scan rate of 20 mV s^{-1} in different CH_3OH electrolyte concentrations.

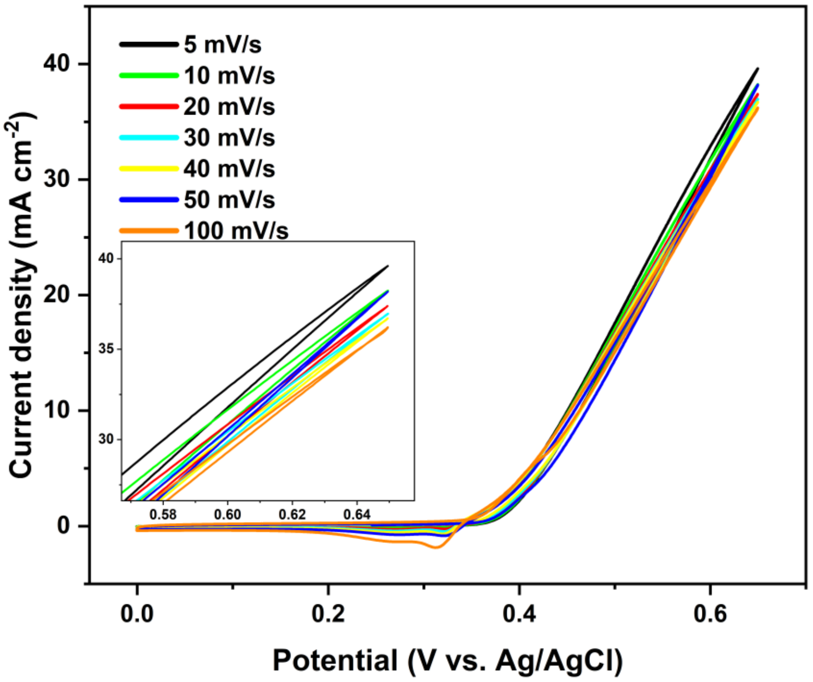

Figure S20. CV curves of Ni/CeLaCuO recorded in a 0.5 M CH_3OH + 1.0 M KOH electrolyte at varying scan rates.

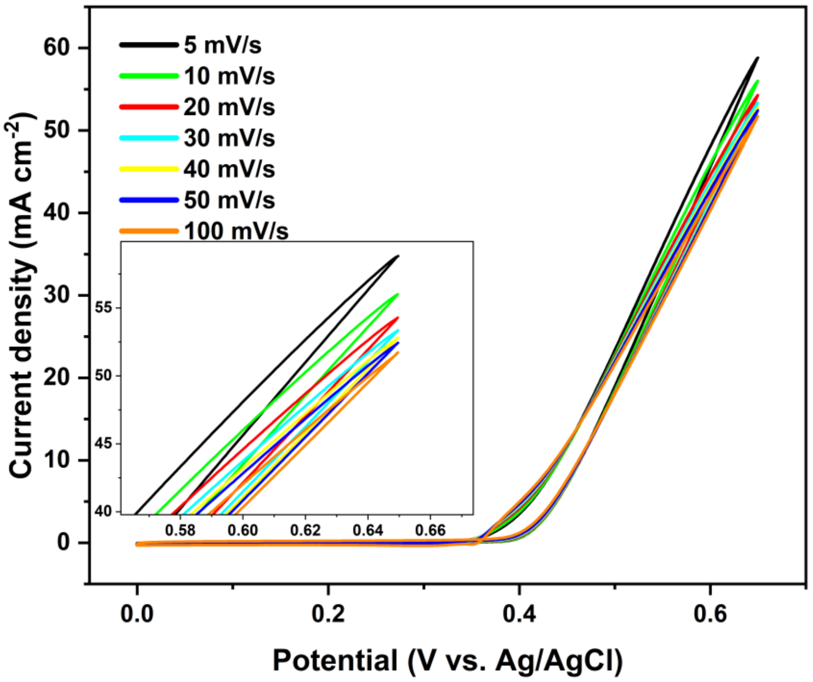

Figure S21. CV curves of Ni/CeLaCuO recorded in a 2.0 M CH_3OH + 1.0 M KOH electrolyte at varying scan rates.

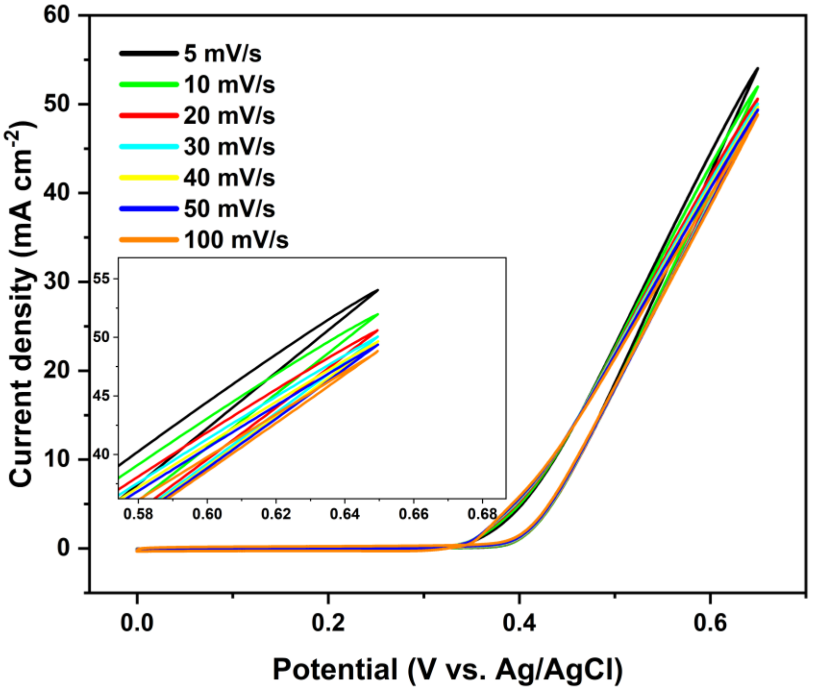

Figure S22. CV curves of Ni/CeLaCuO recorded in a 4.0 M CH_3OH + 1.0 M KOH electrolyte at varying scan rates.

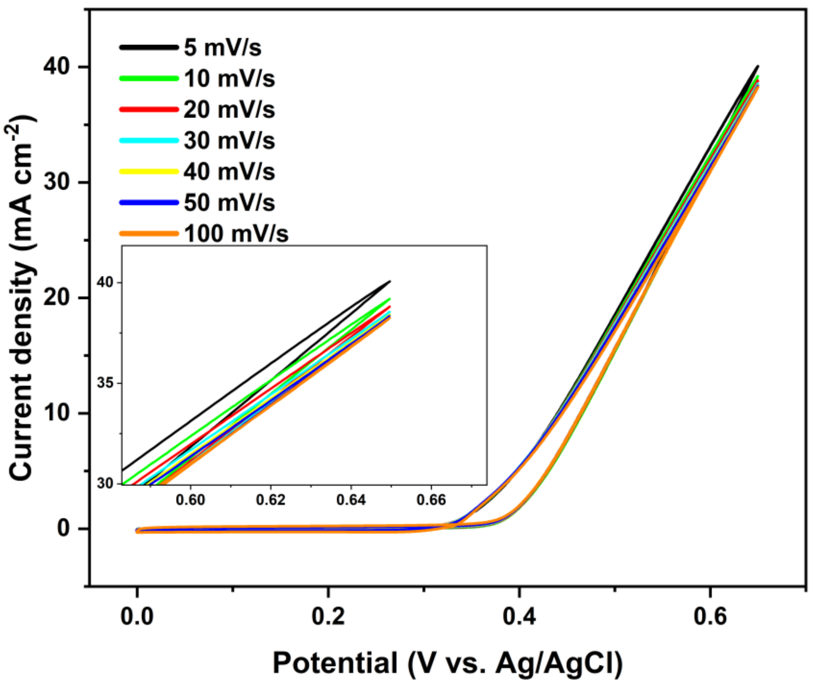

Figure S23. CV curves of Ni/CeLaCuO recorded in a 8.0 M CH_3OH + 1.0 M KOH electrolyte at varying scan rates.

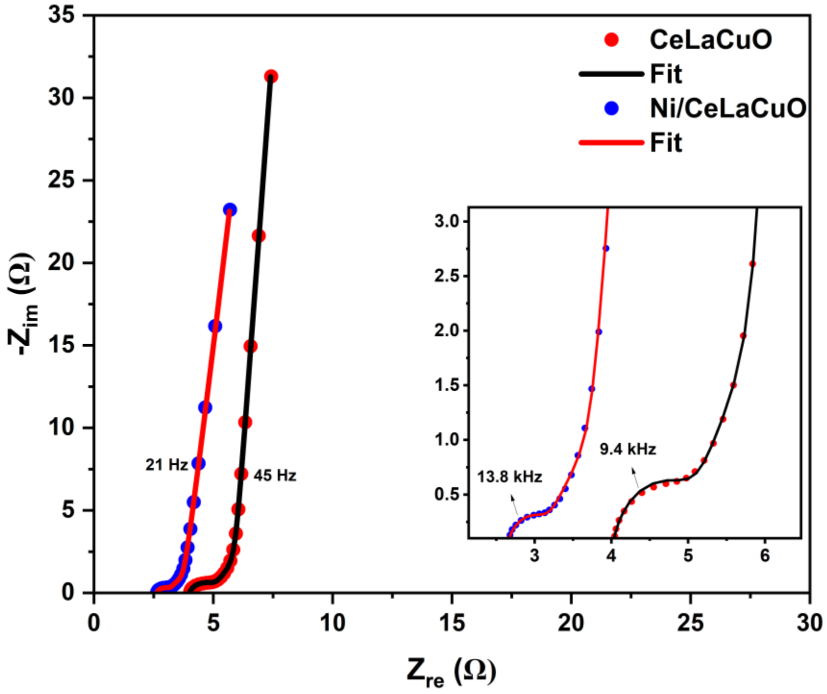

Figure S24. The electrochemical impedance spectroscopy Nyquist plot for CeLaCuO and Ni/CeLaCuO in a 1.0 M KOH + 1.0 M CH_3OH electrolyte at 0 V vs. Ag/AgCl.

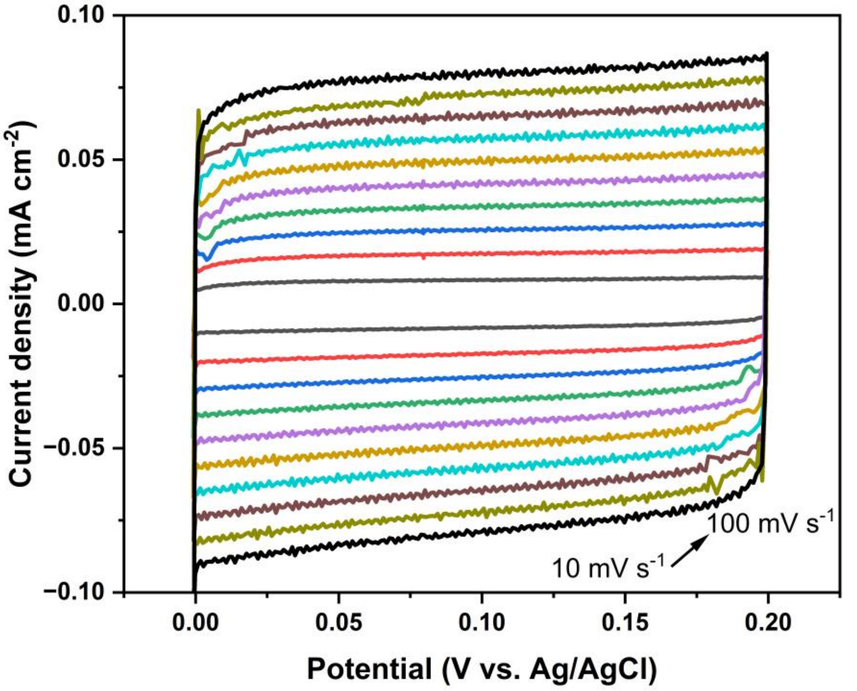

Figure S25. Cyclic voltammetry plots for the CeLaCuO electrocatalyst in the non-faradaic region at scan rates of 10, 20, 30, 40, 50, 60, 70, 80, 90 and 100 mV s^{-1}.

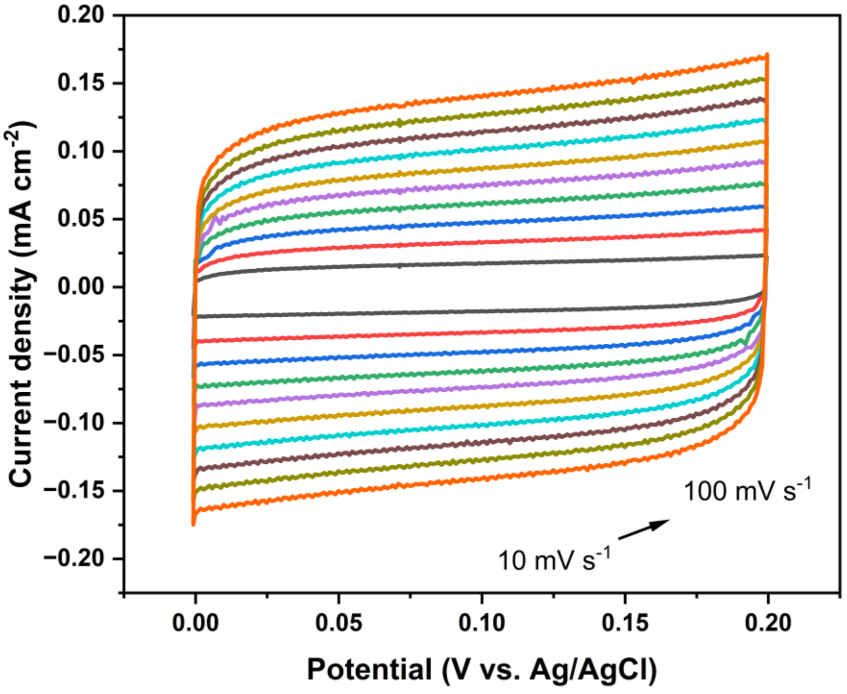

Figure S26. Cyclic voltammetry plots for the Ni/CeLaCuO electrocatalyst in the non-faradaic region at scan rates of 10, 20, 30, 40, 50, 60, 70, 80, 90 and 100 mV s^{-1} before a run of 500 CV cycles in 1.0 M KOH + 1.0 M CH_3OH.

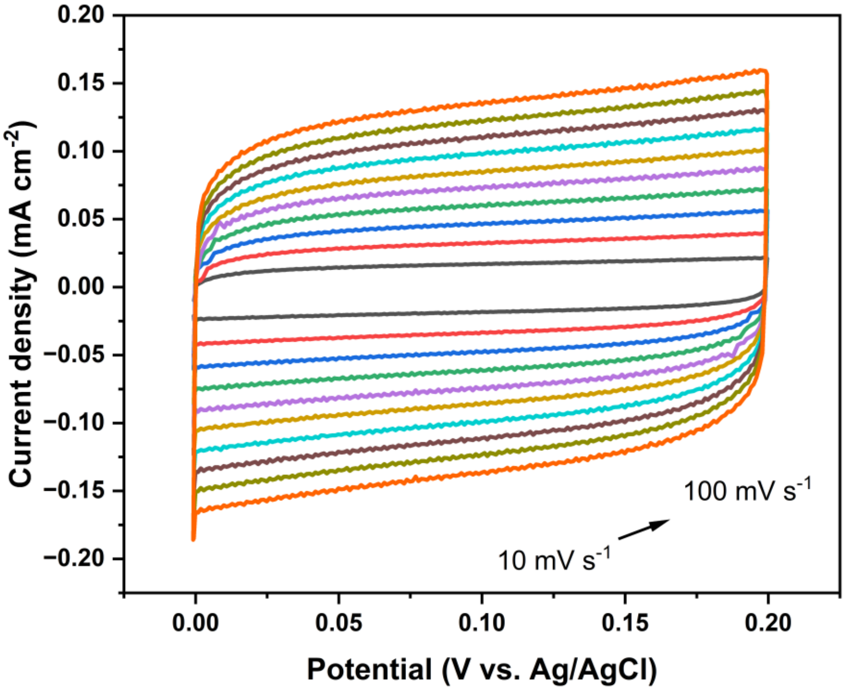

Figure S27. Cyclic voltammetry plots for the Ni/CeLaCuO electrocatalyst in the non-faradaic region at scan rates of 10, 20, 30, 40, 50, 60, 70, 80, 90 and 100 mV s^{-1} after a run of 500 CV cycles in 1.0 M KOH + 1.0 M CH_3OH.

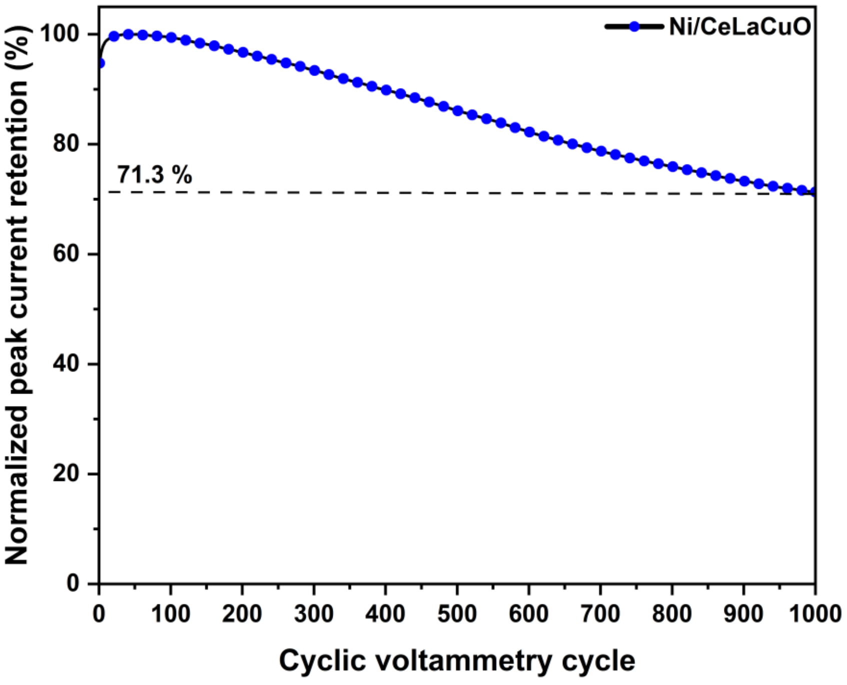

Figure S28. The normalized peak current retention vs. cyclic voltammetry cycles for Ni/CeLaCuO in 1.0 M KOH + 1.0 M CH_3OH at a scan rate of 20 mV s^{-1}.

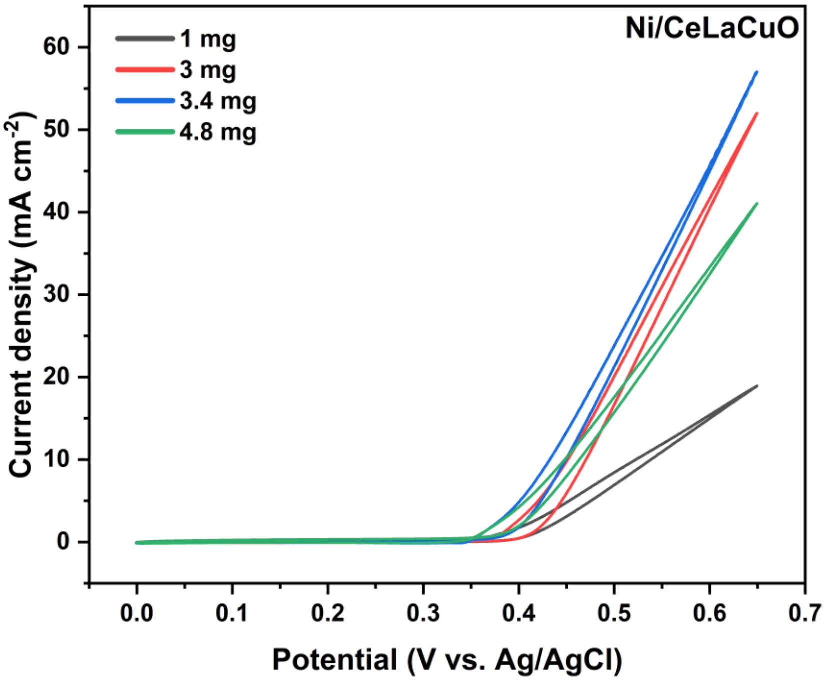

Figure S29. The cyclic voltammetry of different total ink mass loading in 1.0 M KOH + 1.0 M CH_3OH at a scan rate of 20 mV s^{-1}.

Table S2. A comparison of the MOR performance between Ni-incorporated electrocatalysts found in the literature and this work.

Electrocatalyst	Scan Rate (mV s^{-1})	Onset Potential	Peak Potential	Peak Current	Electrolyte	Reference
**Ni/CeLaCuO**	10	0.41 V vs. Ag/AgCl	0.65 V vs. Ag/AgCl	52.3 mA cm^{-2}	1.0 M CH_3OH + 1.0 M KOH	This work
**Ni-NiCu-3**	50	-	-	25.9 mA cm^{-2}	1.0 M CH_3OH + 0.1 M KOH	[5]
**Ni-Co-Mo/CNF**	100	0.22 V vs. Ag/AgCl	0.60 V vs. Ag/AgCl	99.8 mA cm^{-2}	2.0 M CH_3OH + 1.0 M KOH	[6]
**Cu-Ni-S**	-	-	0.65 V vs. Hg/HgO	55.6 mA cm^{-2}	0.5 M CH_3OH + 1.0 M KOH	[7]
**$NiMoO_4$/C**	50	0.45 V vs. Hg/HgO	0.8 V vs. Hg/HgO	49 mA cm^{-2}	2.0 M CH_3OH + 1.0 M KOH	[8]
**Co_1-Ni_4/G**	50	-	-	22.5 mA cm^{-2}	1.0 M CH_3OH + 1.0 M KOH	[9]

References

(1) Caputo, F.; De Nicola, M.; Sienkiewicz, A.; Giovanetti, A.; Bejarano, I.; Licoccia, S.; Traversa, E.; Ghibelli, L. Cerium Oxide Nanoparticles, Combining Antioxidant and UV Shielding Properties, Prevent UV-Induced Cell Damage and Mutagenesis. *Nanoscale* **2015**, *7* (38), 15643–15656. https://doi.org/10.1039/C5NR03767K.

(2) Su, D.; Ford, M.; Wang, G. Mesoporous NiO Crystals with Dominantly Exposed {110} Reactive Facets for Ultrafast Lithium Storage. *Sci Rep* **2012**, *2* (1), 1–7. https://doi.org/10.1038/SREP00924;SUBJMETA.

(3) Li, J.; Li, P.; Li, J.; Tian, Z.; Yu, F. Highly-Dispersed Ni-NiO Nanoparticles Anchored on an SiO2 Support for an Enhanced CO Methanation Performance. *Catalysts 2019, Vol. 9, Page 506* **2019**, *9* (6), 506. https://doi.org/10.3390/CATAL9060506.

(4) Liu, B.; Liu, L. R.; Liu, X. J.; Liu, M. J.; Xiao, Y. S. Variation of Crystal Structure in Nickel Nanoparticles Filled in Carbon Nanotubes. *Materials Science and Technology* **2012**, *28* (11), 1345–1348. https://doi.org/10.1179/1743284712Y.0000000085.

(5) Liu, S.; Sun, Y. Y.; Wu, Y. P.; Wang, Y. J.; Pi, Q.; Li, S.; Li, Y. S.; Li, D. S. Common Strategy: Mounting the Rod-like Ni-Based MOF on Hydrangea-Shaped Nickel Hydroxide for Superior Electrocatalytic Methanol Oxidation Reaction. *ACS Appl Mater Interfaces* **2021**, *13* (22), 26472–26481. https://doi.org/10.1021/ACSAMI.1C04282/SUPPL_FILE/AM1C04282_SI_001.PDF.

(6) Awad, S.; Al-Dies, A. A. M.; Almahdawi, R.; Al-sheqefi, F. U. Y.; Abdel-Hady, E. E. Ni-Co-Mo Nanoparticles as an Efficient Electrocatalyst for Methanol Electro-Oxidation in Alkaline Media. *Polym Adv Technol* **2024**, *35* (2), e6322. https://doi.org/10.1002/PAT.6322;PAGE:STRING:ARTICLE/CHAPTER.

(7) Chinnadurai, D.; Lee, S. J.; Yu, Y.; Nam, S. Y.; Choi, M. Y. Cation Modulation in Dual-Phase Nickel Sulfide Nanospheres by Pulsed Laser Irradiation for Overall Water Splitting and Methanol Oxidation Reaction. *Fuel* **2022**, *320*, 123915. https://doi.org/10.1016/J.FUEL.2022.123915.

(8) Jothi, P. R.; Kannan, S.; Velayutham, G. Enhanced Methanol Electro-Oxidation over in-Situ Carbon and Graphene Supported One Dimensional NiMoO4 Nanorods. *J Power Sources* **2015**, *277*, 350–359. https://doi.org/10.1016/J.JPOWSOUR.2014.11.137.

(9) Sarwar, E.; Noor, T.; Iqbal, N.; Mehmood, Y.; Ahmed, S.; Mehek, R. Effect of Co-Ni Ratio in Graphene Based Bimetallic Electro-Catalyst for Methanol Oxidation. *Fuel Cells* **2018**, *18* (2), 189–194. https://doi.org/10.1002/FUCE.201700143.
